# DNA barcoding, micromorphology and metabolic traits of selected * Ficus* L. (Moraceae) species from Egypt

**DOI:** 10.1186/s12870-024-05683-4

**Published:** 2024-11-13

**Authors:** Marwa M. El-Demerdash, Ashraf S. A. El-Sayed, Samir S. Teleb, Ahmed M. Sadek, Heba. H. Elsehely

**Affiliations:** 1 Botany and Microbiology Department, Faculty of Science, Zagazig University, Zagazig, 44519 Egypt; 2 Botany and Microbiology Department, Faculty of Science (Boys Branch), Al-Azhar University, Cairo, 11884 Egypt; 3 Botany Department, Faculty of Science, Tanta University, Tanta, 31111 Egypt

**Keywords:** DNA barcoding, *Ficus species*, ITS region, Microanatomy, Metabolic traits

## Abstract

The genus *Ficus* of the family Moraceae, is one of the largest genera of angiosperms, with diverse pharmaceutical applications and biological activities. The traditional approaches based on the morphological traits have been frequently implemented for taxonomical identification of the different taxa of *Ficus*, however, encompassing these features are quite laborious, due to the dependence of these phenotypic traits on the environmental conditions. So, authenticating the taxonomical identity of the *Ficus* taxa with molecular barcoding and metabolic profiling, as relatively stable traits, could be a relevant approach for confirming the traditional phenotypic traits of this genus. Nine species of the genus *Ficus* namely *F. amplissima* Sm., *F. benjamina* L. *F. binnendijkii*,* F. drupacea* var. *pubescens*,* F. elastica* Roxb., *F. microcarpa* L., *F. religiosa* L., *F. tinctoria* subsp. *gibbosa* and *F. virens* var. *sublancelata* in Egypt, were selected for this study. From the anatomical features, three species of subsection Urostigma, *F. religiosa*,* F. virens* var. *sublanceolata* have cystoliths on the abaxial layer, whereas in *F. amplissima* it was on the adaxial layer. The UPGMA dendrogram of the studied *Ficus* taxa has been generated from the 21 anatomical characters, categorized the studied taxa into two clusters (I and II) of average distance ~ 3.5, each cluster has been further divided into subclusters I and II. The sub-cluster I includes *F. religiosa*,* F. virens* var. *sublanceolata* and *F. tinctoria* subsp. *gibbosa* were grouped together to subsection Urostigma, while the sub-cluster II of the cluster I includes *F. benjamina* and *F. amplissima*. From the DNA barcoding analysis, three clusters I, II and III were emerged, the cluster I includes *F. benjamina*,* F. binnendjikee*, and *F. amplissima*. The cluster II, *F. virens* var. *sublanceolata* and *F. religiosa* that belong to subsection Urostigma, while, the cluster III includes *F. elastica* and *F. drupacea* var. *pubescens*,* F. microcarpa* that belongs to subsection Conosycea. From the metabolic profiling of *Ficus* species, the major compounds; H-cycloprop-azulen-7-ol, 3,7,11,15-Tetramethyl-2-hexadecen-1-ol, 2-(9-octadecenyloxy), pentadecanoic acid, phytol, sitosterol and 9,12-octadecadienoic acid were the common among the taxa, with an obvious fluctuation, that could be a chemotaxonomic markers for these species of *Ficus*. Based on the metabolic profiling, two distinct clusters I and II were evolved, the cluster I involve *F. elastica*, *F. benjamina*, *F. drupacea* var. *pubescens*, *F. amplissima*, while, the cluster II had *F. tinctoria* subsp. *gibbosa* and *F. religiosa*. The fluctuation on the metabolites of the tested *Ficus* species could be a metabolic fingerprint for each species. So, the delamination of the tested plants based on their anatomical traits was typically matched to the separation based on the ITS sequence analysis.

**Supplementary Information:**

The online version contains supplementary material available at 10.1186/s12870-024-05683-4.

## Introduction

The genus *Ficus* of Moraceae is one the most frequent genera of angiosperms with about 1000 species worldwide [1, 2], that is distributed in Asia, Australia, Africa and America tropical and subtropical regions [3]. The diverse species of *Ficus* inhabits many ecological niches, deciduous, evergreen trees, shrubs, herbs, climbers, and freestanding trees. The *Ficus* species have been considered as exceptionally important food resources, played a crucial role in the ecosystems [4]. *Ficus* has been used frequently for traditional medications, and food sources, fodder, and fuel, hedges or enclosures [4]. The pharmaceutical properties of the genus *Ficus* have been extensively reported with relevant antimicrobial activities [5], antioxidant [6], anticancer, anti-diabetic activities [7, 8], in addition to anti-inflammatory. Unlike the extensive applications of *F. religiosa*,* F. benjamina* [9, 10], few studies about the descriptive pharmacological properties were reported. The different species of *Ficus* have been reported to have diverse pharmaceutically important classes of phytochemicals with various biologically activities [11–13]. The identity of the secondary metabolites in *Ficus* species has been strongly related to the chemotaxonomy of the plant [14].

The main taxonomical features of *Ficus*, relied on the anatomical characteristics of leaf epidermis, shape of epidermal cells, presence of epicuticular waxes [15, 16]. The structure of stomatal complexes has been actually used as valuable diagnostic and taxonomic traits [17–20]. The foliar anatomical features of the studied 25 species of *Ficus* in Nigeria, emphasize the relevance of these features as a key taxonomical tool [16]. The microscopical features of the leaves of *Ficus* provide a relevant tool to identify this genus into subgenera, series, and subseries [21, 22]. Ficus has been divided into 6 subgenera, *Urostigma*, *Pharmacosycea*, *Sycomorus*, *Sycidium*, *Synoecia* and *Ficus* [23]. The subgenus *Ficus* has been divided into sections *Ficus* and *Eriosycea*, while the subgenus *Synoecia* including sections *Rhizocladus* and *Kissosycea* are the main [23]. The subgenus *Sycidium* has two sections *Sycidium* and *Palaeomorphe*, the subgenus *Urostigma* includes sections *Americana*, *Urostigma*, *Malvanthera* and *Galoglychia* [23, 24]. The subgenus and section of *Ficus* is a taxonomically complex section of the genus *Ficus* in which the taxonomical identification remains ambiguous, due to the considerable variations in their morphological characteristics [25, 26]. Taxonomical complexity of the *Ficus* species was attributed to the overlapping morphologies, incomplete genetic differentiation, and emergence of various speciation processes [27].

The genus *Ficus* followed various peculiar ways of evolution, its taxonomy still unclear and confusing due to significant morphological variety and ambiguous borders between groups [28, 29]. The morphological traits of the different species of the genus *Ficus* are greatly varied with the environmental and ecological conditions, so identification of these plants based on their morphological features being a quite bewildering, especially *F. amplissima* Sm, *F. virens* Aiton and *F. elastica* Roxb [24, 30]. Recently, molecular authentication of the different species of the genus *Ficus* depending on their DNA sequence has been extensively used to verify the traditional taxonomical features. DNA barcoding based on the sequence of coding/non-coding genes is a one of the emerged technologies for plant identification due to their efficiency, independence on the environmental conditions, and plant age [4, 31–36]. Recently, DNA barcoding technique is fast and efficient tool to identify the species (interspecies divergence) [37]. In plants, several coding and noncoding regions of chloroplast DNA (*rbcL*, *matK*, *ycf1*, *ycf*5, *trnL*, *rpoc*1, *trnH-psbA*) [38, 39] and internal transcribed spacer (ITS) region of nuclear ribosomal DNA have been authenticated as a potential DNA barcode candidate [40]. The molecular markers for plant identification based on the sequence of a specific DNA segment of the plant genome have been recently remarked with reliable accuracy over the phenotypic traits due to their relative stability, presence in all the plant tissues, independent on the environmental conditions [41–43].

Several taxonomical studies of the genus *Ficus* were reported based on the plant traditional morphological and anatomical features [14, 44–46]. However, in Egypt, few studies integrating the traditional taxonomical features, with the molecular barcoding (ITS sequence) and metabolic pattern of *Ficus* were documented. The common species of *Ficus* in Egypt are *F. drupacea* var. *pubescens*, *F*. *microcarpa*, *F*. *benjamina*, *F. amplissima*, *F*. *virens* var *sublanceolate*, *F*. *religiosa*, *F*. *elastica*,* F*. *binnendijkii*, and *F*. *tinctoria* subsp. *gibbosa*. Several studies recommended the rDNA sequence as the best barcode candidate for closely related plant species [25, 31, 39, 45–49]. Therefore, the objective of this study was to integrate the traditional anatomical features, morphological features, chemotaxonomical traits and DNA barcoding in revising the identification of the *Ficus* species, based on the recent molecular barcoding tools.

## Materials and methods

### Collection of the plant samples

Nine species of the *Ficus* were identified according to [50–53], relied on their anatomical and morphological characterizations. All collected samples were authenticated in reference to the Cairo University Herbarium. Specimens of studied taxa were kept in the Herbarium of Botany and Microbiology Department, Faculty of Science, Zagazig University, Egypt (Table 1). All studied taxa were collected from Orman Botanical Garden, and kindly identified by Dr. Therese Labib, Plant Taxonomist at Orman Botanical Garden in Giza, Egypt. The scientific names of the plants were verified according to the website of the international Plant Names Index: http://www.ipni.org./ipni/query_ipni.html.

**Table 1:** Voucher information of materials used for study

Taxa	Subgenus	Taxonomy section	Subsection*	Deposition Number
Ficus amplissima Sm. *synonames Ficus indica* Wild. *Ficus pseudobenjamina* (Miq.) Miq.*Ficus tjiela* Miq.*Ficus tsiela* Roxb. ex Buch.-Ham. *Ficus pseudotsiela Trimen* Miq.,*Urostigma pseudobenjaminum* Miq. *Urostigma pseudotjiela* Miq.	*UROSTIGMA*	*UROSTIGMA*	*Urostigma*	002155 CF-29, 05–06 -01-19
*Ficus benjamina* L. (Roxb.) Kurz Synonyms: *Urostigma nudum; Ficusbenjamina var. comosa; F. comosa;*	*UROSTIGMA*	*UROSTIGMA*	*Conosycea*	002165 CF-33, 05–06–01–33
*Ficus binnendijkii* Synonyms *Ficus longifolia*	*UROSTIGMA*	*UROSTIGMA*	*Conosycea*	002166 CF − 34, 05–06 -01- 34
*Ficus drupacea* Thunb. var. *pubescens* (Roth) Corner Synonames: Ficus mysorensis var.pubescens	*UROSTIGMA*	*UROSTIGMA*	*Conosycea*	002213 CF- 81, 05-06-03-24
*Ficus elastica* Roxb. ex Hornem Synonyms: *Ficus cordata; F. skytinodermis; F. taeda; Urostigma circumscissum;*	*UROSTIGMA*	*UROSTIGMA*	*Conosycea*	002165 CF − 33, 05–06-02-08
*Ficus microcarpa* L. f. Synonyms: *Ficus amblyphylla; F.cairnsii; F. condaravia; F. retusiformis; F. rubra; Urostigma amblyphyllum*	*UROSTIGMA*	*UROSTIGMA*	*Conosycea*	002193 CF-61, 05- 06- 03–04
*Ficus religiosa* L. Synonyms: *Urostigma religiosum.*	*UROSTIGMA*	*UROSTIGMA*	*Urostigma*	002187 CF 86, 05 -06-04 -03
*Ficus tinctoria* G. Forst. subsp. *gibbosa* (Blume) Corner* Synonyms: *Ficus gibbosa; F. cuspidifera; F. gibbosa var. cuspidifera; F. gibbosa var. parasitica*	*SYCIDIUM*	*PALAEOMORPHE*	NA	002209 CF -77, 05–06-03-20
*Ficus virens var sublancelate*	*UROSTIGMA*	*UROSTIGMA*	*Urostigma*	002235CF-103, 05- 06-04-20

*Taxonomy of Ficus according to Berg and Corner [23]

### Morphological characterization and microanatomy of plants

The plant leaves were collected, washed, then fixed in formalin, glacial acetic acid and ethyl alcohol 70% (5:5:90 v/v), followed by sectioning to 12–15 μm by microtome, then embedded in paraffin wax, stained with safranin [54], prior to microscopical analysis [55]. The plant sections were photographed by the light microscope (Optika, MA88-900 digital camera). The anatomical features of the investigated sections of plants were assessed based on the reference morphological keys [50]. The data of morphological traits of the analyzed leaves were scored, coded to construct the numerical data matrix, and analyzed by the PAleontological STatistics (PAST) software (version 6.0) [56].

The epidermal structures of the leaves were observed by scanning electron microscope (SEM), the sections of the adaxial and abaxial surfaces of the leaves were placed directly on double-sided tape. The specimens were coated with thin layer gold palladium before being examined by SEM placed with carbon paste on an AL-stub and coated with gold up to 400 °A in thickness (JFC-1100 E). The electron microscope utilized for the investigations was a Jeol JSM-5300 operating at an energy level ranging from 15 to 20 keV (Electron Microscope Unit, Faculty of Science, Alexandria University, Egypt).

### DNA isolation, PCR amplification and sequencing

The plants were molecularly identified based on the sequence of the ITS regions [33–38]. The plant genomic DNA was extracted by Cetyl trimethyl ammonium bromide (CTAB) lysis buffer [57]. The plant tissues (0.1 g of fresh weight) was minced in liquid nitrogen, amended with 500 µl of CTAB buffer, vortex, and centrifugation for 10 min at 10000 xg (Sigma Laborzentrifugen GmbH, Germany). The supernatant was amended with equal volume of chloroform, shaking vigorously, then centrifuged at 15000 xg for 15 min. Double volume of absolute ethanol was amended to the supernatant, then incubated in -20°C for 30 min, the DNA was pelleted by 10000 xg centrifugation for 10 min, dispensed in 50 µl of sterile distilled water, their purity and concentration of the DNA were tested by agarose gel (1.5%), with 1 kb DNA ladder (Cat.# PG010-55DI, Thermo Fisher Scientific). Plant gDNA was used as a PCR template, with the universal primers ITS1 5’-TCCGTAGGTGAACCTGCGG-3’, ITS4 5’-TCCTCCGCTTATTGA TATGC-3’ [58]. The PCR reaction contains 10 µl of 2× i-Taq™ master mixture (Cat.# 25–027, Boca Scientific Inc. MA, USA), 1 µl gDNA, 1 µl of 10 pmol of each primer, and completed to 20 µl with nuclease-free water. The PCR conditions were adjusted for initial denaturation 94 °C for 4 min, denaturation 94 °C for 30 s, annealing 51 °C for 30 s, and extension 72 °C for 1 min for 35 cycles, and final extension for 5 min at 72 °C (T100 Thermal Cycler BioRad). The PCR amplicons were analyzed by 1.5% agarose gel, and the ITS regions were sequenced, non-redundantly BLAST, and aligned by Clustal W muscle algorithm with MEGA X software [59]. The phylogeny of the ITS sequences was analyzed by the neighbor-joining method [60]. The ITS sequences were trimmed by BioEdit the length and GC ratio were determined by Endmemo software package [61]. The positions containing gaps were excluded and the values of internal branches of NJ tree were assessed by 100 bootstrap replicates. The transition/ transversion ratio ti/tv was determined by the formula R=[A*G*k1 + T*C*k2]/ [(A + G)*(T + C)] [62]. The number of nucleotide substitutions per site between sequences was estimated. The sequences were analyzed with DnaSP software [63] to estimate Fu and Li’s D* and F* values [64].

### Metabolic profiling of the experimental plants by GC-MS analysis

The fresh, healthy leaves from the gathered specimens were shade dried for two weeks before being crushed to a fine powder with a household blender. The leaves that had been ground into a powder (10 g) were macerated in methanol (1:5 w/v) in firmly sealed conical flasks at room temperature for 72 h [65]. The extract was filtered, centrifuged at 5000 rpm for 10 min, and the supernatant was collected and the solvent was evaporated to dryness, then dissolved in 1 ml methanol, in tightly sealed dark vials at 4 °C till use. Three technical replicates were used for the metabolomic analysis. The chemical components of each the extract were analyzed with the Trace GC1310-ISQ mass spectrometer (Thermo Scientific, USA) with a direct TG-5MS capillary column (30 m x 0.25 mm x 0.25 μm film thickness). The temperature of the column oven was first held at 50 °C, then increased by 5 °C/min to 230 °C, held for 2 min, and then increased by 30 °C/min to the ultimate temperature of 290 °C, held for another 2 min. The temperatures of the injector and MS transfer line were maintained at 250 °C and 260 °C, respectively; a 1 ml/min flow rate of helium was utilized as the carrier gas. The samples were injected to an Autosampler AS1300 coupled with GC in the split mode, with a solvent delay of 3 min. EI mass spectra were acquired in full scan mode at 70 eV ionization voltages of m/z 40-1000, in full scan mode, with ion source temperature at 200 °C. The chemical structure of the resolved spectral mass was identified compared to their retention times and mass spectra referencing to the NIST mass spectral databases.

### Clustering analysis

The features were represented as present (1) or absent (0), to form a binary matrix for all the samples. Data analysis was performed using the NTSYS-pc software version 2.1 [66]. Jaccard’s similarity coefficients were used to generate a dendrogram using Unweighted Pair Group Method with Arithmetic Average (UPGMA) [67] and relationships between the samples were represented in the dendrogram.

## Results and discussion

### Morphology and Microanatomy of the tested* Ficus* species

Leaf morphological characters such as leaf arrangement, shape of lamina, leaf apex and margin are often used as morphological traits for species identification of *Ficus* (Jangam et al., 2017). The studied taxa showed a considerable variable macromophological features like leaf shape, leaf base, that were ovate-elliptic with cuneate base in *F. tinctoria* subsp. *gibbosa*, elliptic with acute to round base in *F. benjamina*, ovate-lanceolate with subcordate base in *F. amplissima* and *F. binnendijkii*, elliptic to ovate-elliptic with acute to cuneate in *F. virens* var. *sublanceolata*, obovate-elliptic with rounded base in *F. drupacea* var. *pubescens*. All the studied species have entire leaf margin. All the species have a spiral leaf arrangement, except *F. benjamina* has an alternate leaves. The leaf apex shows variation, caudate restricted only in *F. religiosa*, acuminate in most of studied taxa such as *F. elastica*, *F. benjamina*, *F. tinctoria* subsp. *gibbosa* and *F. binnendijkii*, and acute in *F. drupacea* var. *pubescens*. The morphological view of the studied species of *Ficus* were shown in Fig. S1.

**Fig. 1:**
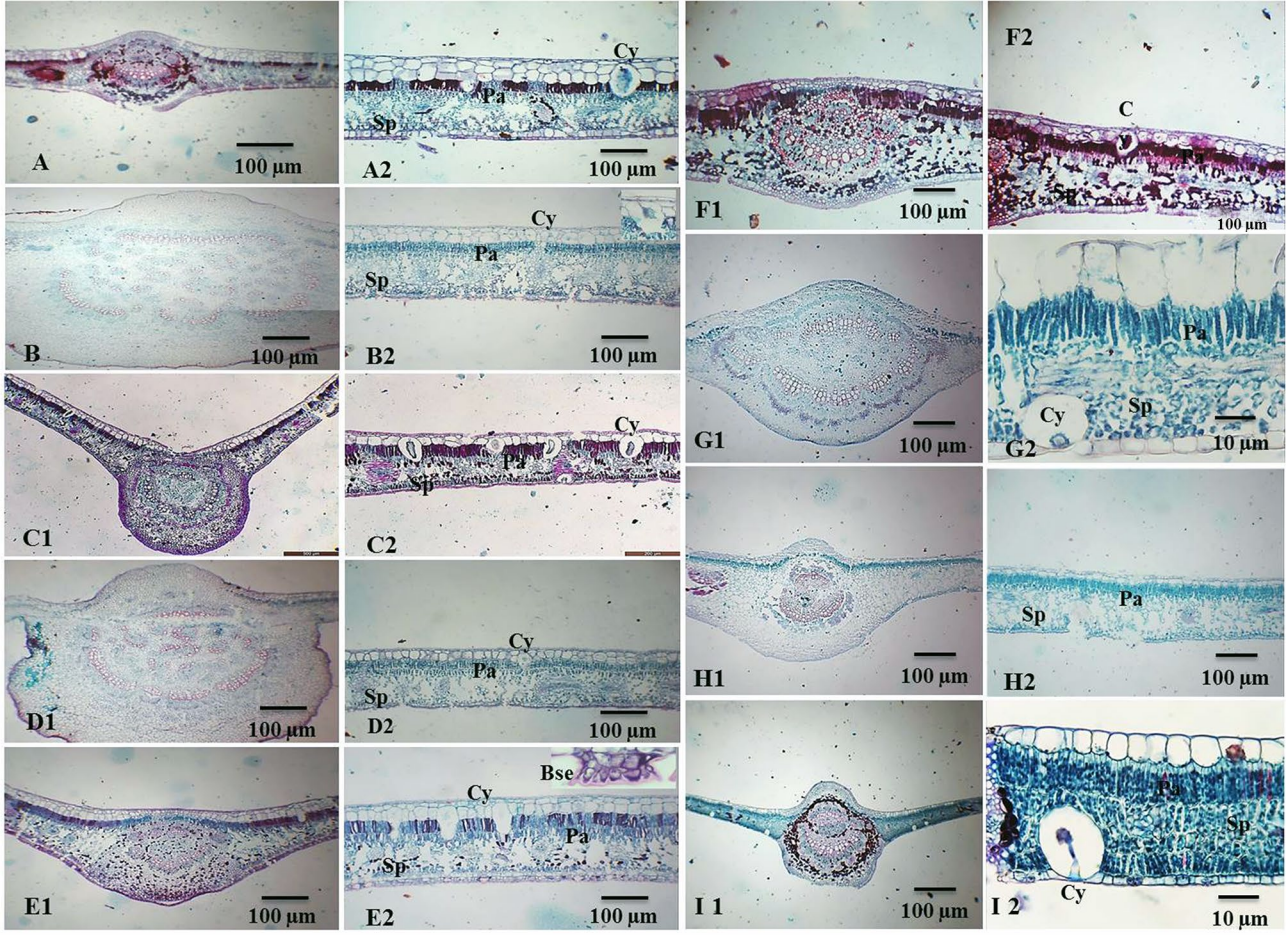
Blade transections of *Ficus* species as follows, *F. amplissima* (**A1**-**A2**), *F. benjamina* (**B1**-**B2**), *F. binnendijkii* (**C1**-**C2**), *F. drupacea* v. *pubescens* (**D1**-**D2**), *F. elasctica* (**E1**-**E2**), *F. microcarpa* (**F1**-**F2**), *F. religiosa* (**G1**-**G2**), *F. tinctoria* subsp. *gibbosa* (**H1**-**H2**) and *F. virens* v. *sublanceolata* (**I1**-**I2**). Cy.= Cystolith; Pa.=Palisade; Sp.=Spongy; Sc.=Stone cell

Leaf anatomy has been reported as a key tool in plant taxonomy over the years [68, 69]. The anatomical features of the leaves of the experimented plants namely *F. amplissima*,* Ficus benjamina*,* Ficus binnendijkii*, *Ficus drupacea* var. *pubescens*,* Ficus elastica*,* F. microcarpa*,* F. religiosa*,* F. tinctoria* subsp. *gibbosa* and *F. virens* var. *sublanceolata* showed high similarity in blade transection structure (Fig. 1, A-I). Most midrib of studied taxa are rounded in shape except in *F. elastica* was arc shaped (Fig. 1, E1, E2) or line shape in in *F. microcarpa* (Fig. 1, A1, A2, B1, B2, F1, F2). The main vascular bundle mostly arranged in cycle or crescent shape and collateral in type. The medullary vascular bundle generally present while phloem patches in midrib center mostly absent found only in *F. benjamina*. Ground tissue mainly contains parenchymatous, collenchyma and sclerenchyma tissues. Stone cells are present in *F. tinctoria* subsp. *gibbosa* only (Fig. 1, H1, H2). Mesophyll clearly differentiated into palisade and spongy parenchyma also may isolateral or dorsiventral and non-continuous in midrib. Mesophyll thickness at most species and thin in *F. religiosa* and *F. virens* var. *sublanceolata* (Fig. 1, G1, I1, I2).

**Fig. 2:**
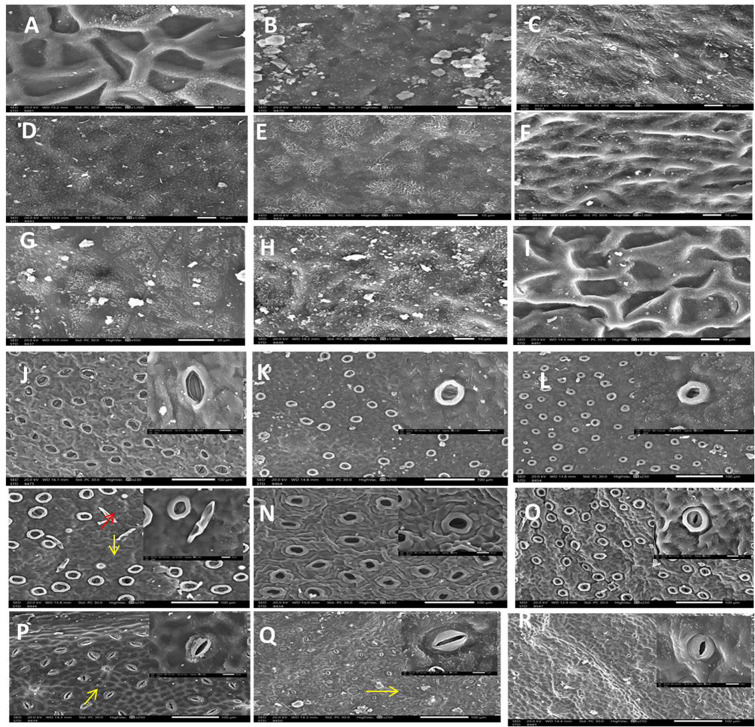
Adaxial surface of leaves *F. amplissima* (**A**), *F. bejamina* (**B**), *F. binnendijkii* (**C**), *F. drupacea* var. *pubescens* (**D**), *F. elastica* (**E**), *F. microcarpa* (**F**), *F. reilglosa* (**G**), *F. tinctoria* subsp. *gibbosa* (**H**), *f. virens v. sublanceolata* (**I**), *F. amplissima* (**J**), *F. bejamina* (**K**), *F. binnendjikee* (**L**), *F. drupacea* (**M**), *F. elastica* (**N**), *F. microcarpa* (**O**), *F. reilglosa* (**P**), *F. tinctoria* subsp.*gibbosa*, (**Q**), *F. virens* v. *sublanceolata* (**R**). The scale bar of the figures are 100 μm, with 250 x amplification. The scale bar of the onset figures for each one was 10 μm with 1500 x amplification. Yellow arrows refers to hyadathode, Red arrow refers to trichomes

Cuticle surface may thin or thick and mostly smooth. Epidermal cell shape cubic, oblong or babble. Upper epidermis thickness (10–17 μm) and spacious in species with no hypodermis such as in *F. religiosa* (Fig. 1, E2). *F. virens* var. *sublanceolata* or thin (2–7 μm), while lower epidermis always thin. The presence of uniseriate epidermal cell in upper and lower epidermis characterized all studied taxa except in *F. elastica* (Fig. 1, E2) multi epidermis in which can be used to separate these taxa from others. This character is practically significant because the taxa are in different taxonomic ranks according to previous classifications systems [70–73]. Multiple epidermis and hypodermis functions as water storage tissue, thick epidermis considered xeromorphic plant character [74]. The epidermis is wide and spacious in species with no hypodermis such as *F. religiosa*,* F. virens* var. *sublanceolata* (Fig. 1, G1, G2-I2), but hypodermis mostly present with 1–4 layers adaxially and one layer abaxially. Several species of the Moraceae are distinguished by the presence of cystoliths [75]. The appearance and position of calcium oxalate or calcium carbonate crystals “cystolith”, is a distinctive and valuable in plant taxonomy [66]. Subsection Conosycea comprises seven species that exhibit cystoliths, which may be present on the adaxial layer or in both epidermal layers [76]. Cystolith mainly present adaxial layer in *F. benjamina*,* F. binnendijkii*, *F. drupacea* v. *pubescens*, *F. elastica*,* F. microcarpa* under subsection Conosycea (Fig. 1, C2, D2, E2, F2). Among three species under subsection Urostigma *F. religiosa*, *F. virens* var. *sublanceolata* (Fig. 1, G1, G2, I1, I2) have cystoliths on the abaxial layer, whereas in *F. amplissima* they are on the adaxial layer [77, 78]. In this study, the transverse-section through the leaf mid-vein of *F. binnendijkii*, *F. drupacea* var. *pubescens*,* F. microcarpa*,* F. religiosa*,* F. tinctoria* subsp. *gibbosa* reported the presence of characteristic druse crystals (Fig. 1, C1, D1, E1, F1, G1). The presence of these druse crystals is significant in plant taxonomy due to the fact that they present in a variety of morphological forms, such as prisms, styloids, raphides, and crystal sand, which differ among plant species [79]. Previous studies also reported the presence of druse crystals in the leaves of other *Ficus* species [80]. Calcium crystals are found in every plant and are generally recognized for their ability to defend plants against herbivores [76]. Leaf epidermal features of *Ficus* have a number of advantages as taxonomic markers, these features exhibit an interesting interspecific variation with great significance for plant identification [80]. The epicuticular wax was reported to cover the cuticle in most studied taxa in different forms of the cuticle (Fig. 2, A-R). SEM observations of the adaxial epidermal leaf surface showed that outer surface of the cuticle is smooth to weakly undulate in most species (Fig. 2, B, C,D, E,G, H) and unusual regular with exaggeratedly thick rugae in *F. amplissima*,* F. microcarpa* and *F. virens* var. *sublanceolata* (Fig. 2, A, F,I). Wax crystalloids are overall distributed in the entire leaf surface in *F. benjamina*, both granules and threads wax look like present in *F. elastica.* Some wax crusts are seen in the leaf adaxial surface of *F. religiosa* and *F. tinctoria* subsp. *gibbosa* (Fig. 2, G, H), that has been consistent with those reported [79, 80] that indicate presence of wax on the adaxial surface of *Ficus* species in form of a crust, film flakes, a fissured layer, platelets and rosettes of platelets, granules, or lumpy. Recrystallization studies found that environmental conditions predominantly control the wax structure. A change in wax structure may indicate a harmful impact of the environmental conditions [77]. The taxonomic significance of trichomes in angiosperm has been frequently documented [78]. The studied taxa of *Ficus* are belongs to glabrous group which include all studied taxa except *F. durpacea* var. *pubescens* which is hairy having unicellular, non-glandular, simple hairs with narrow apiculate tips on abaxial surface, but not on the adaxial leaf surfaces (Fig. 2, M). The taxonomic value of trichomes on the current study was greatly limited by their absence in 8 out of the 9 species of the studied *Ficus* species. Also, two types of laminar hyadathode was reported forming a warts and stomata in abaxial surface of leaves but in other taxa such as *F. drupacea* var. *pubescens*,* F. religiosa*,* F. tinctoria* subsp. *gibbosa* was distinguished forming warts (Fig. 2, K, L,M, P,Q). The presence of hydathodes has been reported in thirty-four families of dicotyledons [50] and in taxa of *Ficus* species [16] as well as, the hyadathode has been reported in 34 taxa on the abaxial leaf surface [21]. The leaves of *Ficus* are usually hypostomatic, that being matched with those previously reported [16, 21]. The presence of a stomatal ledge character has been detected in about 80% of the studied taxa [16]. The stomata were surrounded with a cuticular thickening that with a characteristic rim and located deeply sunken in *F. amplissima*,* F. bejamina*,* F. binnendjikee*,* F. drupacea* var. *pubescens*,* F. elastica*, and *F. microcarpa* (Fig. 2, J-O). Stomata with more elliptic guard cell outline and located at level of the others epidermis cells in *F. religiosa*,* F. tinctoria* subsp. *gibbosa*,* F. virens* var. *sublanceolata* (Fig. 2, P, Q.R).

**Fig. 3:**
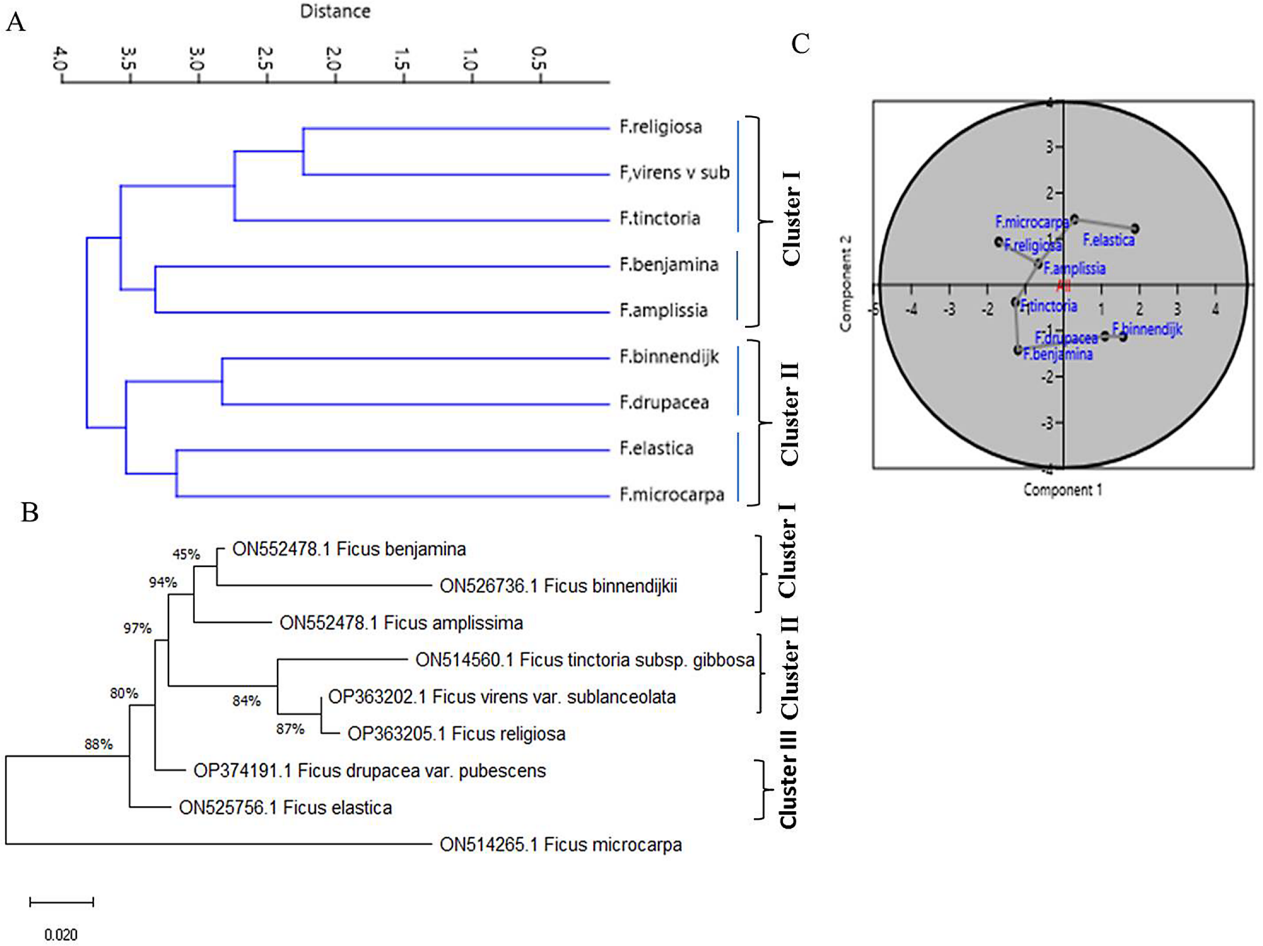
UPGMA analysis (**A**) and PCA (**B**) analysis of the experimented *Ficus* species, based on their anatomical characters. (**C**), The phylogenetic analysis of the ITS sequence of the experimented *Ficus* species using Tamura Maximum Likelihood method

The UPGMA dendrogram of the studied taxa of *Ficus* has been generated from the 21 anatomical characters (Table S1), categorized the all studied taxa into two major clusters I and II with an average taxonomic distance of about 3.5, each cluster has been divided into the clusters I and II (Fig. 3). The sub-cluster I includes *F. religiosa*, *F. virens* var. *sublanceolata*, and *F. tinctoria* subsp. *gibbosa* were grouped together belongs to subgenus Urostigma. The sub-cluster II of the cluster I includes *F. benjamina* and *F. amplissima* (Fig. 3A). The *F. benjamina* belongs to the subsection Conosycea, while, *F. religiosa*,* F. virens* var. *sublanceolata* and *F. amplissima* were belong to subsection Urostigma [23, 25]. The cluster II includes *F. binnendjikee* and *F. drupacea* var. *pubescens* (sub-cluster I) and *F. microcarpa* (sub-cluster II) belongs to the subsection Conosycea that being coincident with the classification [23].

### DNA barcoding and phylogenetic analysis

The studied *Ficus* species were further taxonomically characterized based on their ITS sequences. From the phylogenetic analysis based on the sequence of ITS region (Fig. 3B), three clusters were emerged Cluster I and II and III. The cluster I includes *F. benjamina*,* F. binnendjikee*,* F. amplissima.* The cluster II includes *F. religiosa*, with the same root belongs to subsection Urostigma. *F. tinctoria* subsp. *gibbosa* belong to Palaeomorphe section has been separated as distinct taxa. The cluster III includes *F. elastica* and *F. drupacea* var. *pubescens*, while, *F. microcarpa* belongs to subsection *Conosycea*. Practically, the delamination of the tested plants based on their anatomical traits was matched to the ITS sequence analysis separation.

**Fig. 4:**
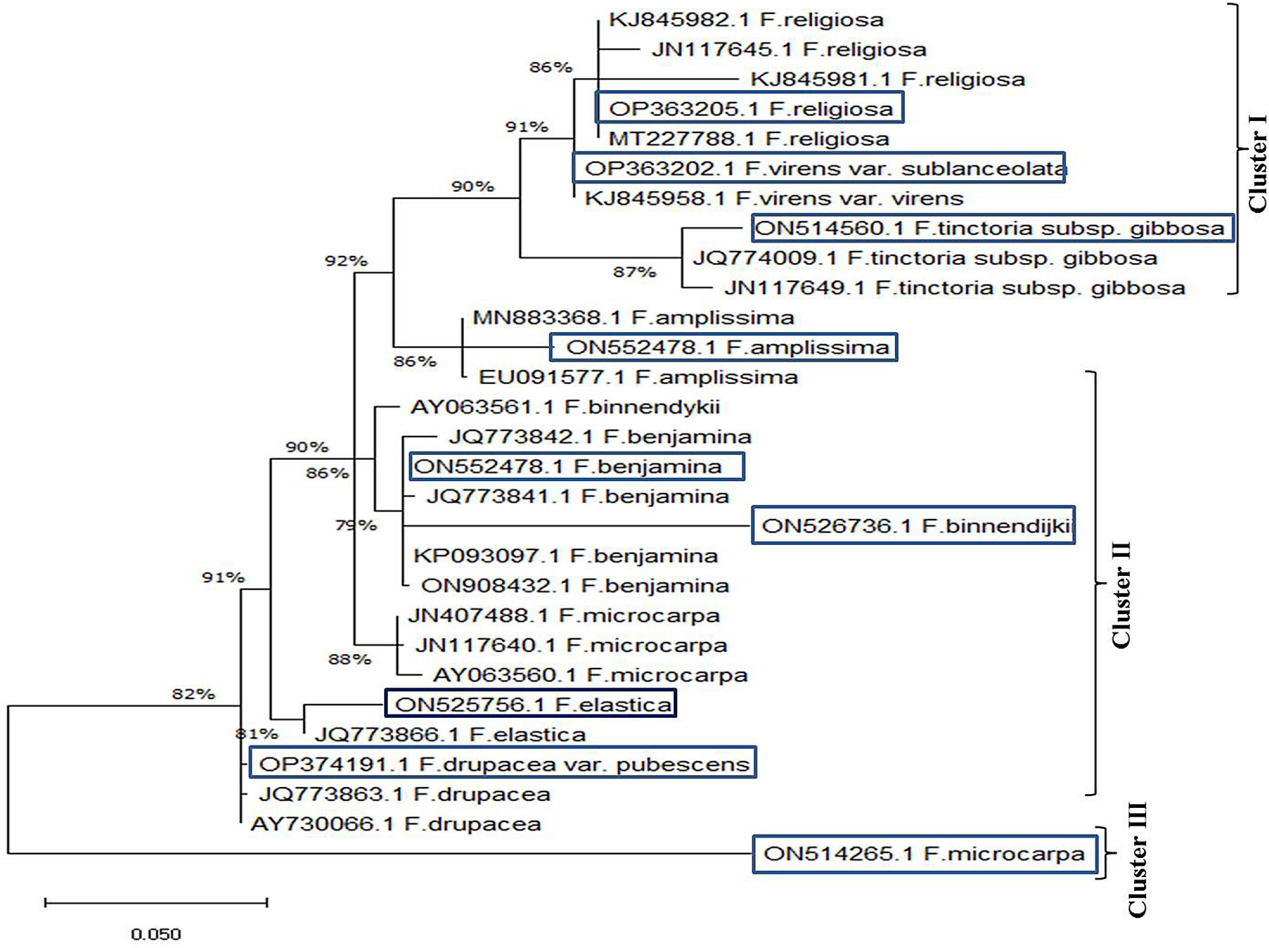
Neighbor-joining relatedness of the eight experimented *Ficus* species based on the ITS sequence with the genbank deposited ITS sequences of the different *Ficus* species

The ITS sequence of the rDNA region has been used to determine the taxonomical identity of the experimental plants. The sequence of the ITS region has been used as a universal phylogenetic marker for differentiating plant species [81]. The ITS sequence has been frequently authenticated for inter-specific divergence of *Ficus* species [82–88]. The plant genomic DNA were used as PCR primer for amplification of the ITS regions. From the PCR amplicons, the size of DNA of the plants was estimated by about 600–700 bp (Fig. 4). The PCR amplicons were sequenced and non-redundantly BLAST searched on the NCBI database. According to the Neighbor-Joining (NJ) method, the studied *Ficus* taxa was categorized into three clusters I, II and III, with the root value 0.05 and similarity ratio ranged from 86 to 92%, with zero E-value. The ITS sequences of the studied taxa of *Ficus* were deposited on the database with the accession numbers as listed on the Table 2. The cluster I include the *F. religiosa*, *F. virens* var *sublancelata* and F. *tinctora* subsp. *gibbosa*. The cluster II includes *F. amplissima*, *F. benjamina*, *F. binnendijkii*, while, the cluster III includes *F. drupacea* var. *pubescens* and *F. microcarpa*. Apparently, based on the ITS sequences, the taxa *F*. *amplissima* was in the middle of the cluster I (*F. religiosa*) and cluster II (*F. benjamina*) are belong to the section *Urostigma*. From the phylogenetic analysis, *F. religiosa*, *F*. *virens* var. *sublanceolata* and *F. tinctora* subsp. *gibbosa*, were belonged to the cluster I that displaying about 86 to 91% similarity with *F. religiosa* KJ845982.1, JN117645.1, KJ845981.1. The cluster II includes *F. benjamina*, *F. amplissima* and *F*. *binnendijkii* that being closely similar to *F. benjamina* KP093097.1, JQ773842.1, EU091577.1, KP093097.1 and ON908432.1. The cluster III includes *F. elastica* ON525756.1, *F. durpacea* var. *pubescens* OP374191.1 that being closely related to the *F. elastica* JQ773866.1, *F. durpacea* var. *pubescens* JQ773863.1, and AY730066.1. The molecular taxonomical barcoding based on the sequence of the ITS region being matched with the traditional taxonomy based on the morphological and anatomical traits. Similarly, molecular identification of the *Ficus* species based on their ITS sequences have been used to authenticates the traditional taxonomical results [89].

**Table 2:** Accession number, length and GC content of the ribosomal DNA sequence of the studied taxa

Scientific Name	NBCI accession no	Length bp	GC%
*Ficus amplissima*	ON552478	722 bp	63.33
*Ficus benjamina*	OP597689	344 bp	62.5
*Ficus binnendijkii*	ON526736	718 bp	60.44
*Ficus drupacea* var. *pubescens*	OP374191	631 bp	65.13
*Ficus elastica*	ON525756	720 bp	63.88
*Ficus microcarpa*	ON514265	669 bp	62.03
*Ficus religiosa*	OP363205	666 bp	63.06
*Ficus tinctoria* subsp. *gibbosa*	ON514560	727 bp	62.17
*Ficus virens* var. *sublancelate*	OP363202	664 bp	63.40

The sequences of the ITS region showing the GC content of *Ficus* species studied have been shown in Table 3. The size of the ITS sequences were varied from 344 to 727 bp in *F. benjamina* and *F. tinctoria* subsp. *gibbosa*, respectively. The GC contents were ranged from 60.44 to 63.88% in *F. binnendijkii* and *F. elastica*, respectively. The rates of different transitional substitutions are shown in bold and those of transversionsal substitutions were shown in italics. The nucleotide frequencies were 20.24% (A), 17.03% (T/U), 31.87% (C), and 30.86% (G). The ratios of transition/transversion for purines and pyrimidines are k1 = 2.792 and k2 = 2.424, respectively, and *R* = 1.224 represents the transition/transversion bias, while, R=[A*G*k1 + T*C*k2]/[(A + G)*(T + C)]. This analysis involved 9 nucleotide sequences, and the included codon positions were 1st + 2nd + 3rd + Noncoding. There were a total of 762 positions in the final dataset (Table 4). The nucleotide composition ratio was 20.8, 18.83, 29.6, and 30.6% for A, T, C, and G, respectively, that being similar to those reported in *Quercus* spp [90]. Consistently, the size of the ITS region in Wheat was 597–605 bp and in Barley was 595–598 bp [91]. The ITS region on Asteraceae family were spanned between 650 and 850 bp with average nucleotide frequency A (25%), T (24%), C (26%), and G (25%), and average GC content 51% and AT 49% [92]. The mean size of *F. carica* ITS was 697 bp and its composition was 19.7% [93]. The ITS1-5.8 S-ITS2 sequence of *Chili* reveals the nuclear frequencies of 18.8% (A), 17.5% (T), 33.9% (C), 34.9% (G) and 29.6% (A) [82]. The ITS region in *Coniferales*,* Cycadales*,* Ginkgoales* and *Gnetales* was ranged between 575 and 700 bp in angiosperms and between 975 and 3125 bp in gymnosperm [82]. The genetic diversity of *Phoenix dactylifera* based on the ITS level was about 2% in the overall data set. Our findings were comparable to those observed in *Quercus suber*,* Trojana* [92], *Glycine max*, and *Tylosema esculentum* [91].The transition/ transversion ratio R of the studied *Ficus* spp was 1.22 in the entire ITS region that is lower than that the entire ITS region in Tunisian cultivars of date palm (ti/tv = 4.375) [87] Asteraceae (ti/tv = 1.43) [93], and *Capsicum* sp (ti/tv = 3.746) [94].

**Table 3:** Maximum Composite Likelihood Estimate of the pattern of Nucleotide Substitution

	A	T	C	G
**A**	-	*3.69*	*6.91*	**18.69**
**T**	*4.39*	-	**16.75**	*6.69*
**C**	*4.39*	**8.95**	-	*6.69*
**G**	**12.25**	*3.69*	*6.91*	-

Each entry shows the probability of substitution (r) from one base (row) to another base (column). For simplicity, the sum of r values is made equal to 100. Rates of different transitional substitutions are shown in **bold** and those of transversionsal substitutions are shown in *italics*. The nucleotide frequencies are 20.24% (A), 17.03% (T/U), 31.87% (C), and 30.86% (G). The transition/transversion rate ratios are *k*_*1*_ = 2.792 (purines) and *k*_*2*_ = 2.424 (pyrimidines). The overall transition/transversion bias is *R* = 1.224, where *R* = [A*G*k_*1*_ + T*C*k_*2*_]/[(A + G)*(T + C)]. This analysis involved 9 nucleotide sequences. Codon positions included were 1st + 2nd + 3rd + Noncoding. There were a total of 762 positions in the final dataset. Evolutionary analyses were conducted in MEGA X

**Table 4:** Results from Tajima’s Neutrality Test

m	S	*p*_s_	Θ	π	D
9	217	0.284777	0.104780	0.067038	-1.872629

NOTE. This analysis involved 9 nucleotide sequences. Codon positions included were 1st + 2nd + 3rd + Noncoding. All ambiguous positions were removed for each sequence pair (pairwise deletion option). There were a total of 762 positions in the final dataset. Evolutionary analyses were conducted in MEGA X

### Selective neutrality tests

The selective neutrality test for the comparative analysis of DNA sequences among species is one of the highly effective method for identifying the evolutionary domains in particular gene regions and for elucidating significant aspects of the evolutionary history of a given species. The neutrality test of the experimental plants was used to assess the trend of plant diversity. The sequence of nuclear ribosomal DNA differed significantly from the neutral balancing model. The selective neutrality for the detected variations was tested by Tajima [93] and Fu and Li’s [64] models to assess the null hypothesis. Tajima D is -1.872629 Fu and Li′s D* test statistic: − 0.98694. Statistical significant *p* < 0.10 Fu and Li′s F* test statistic: -1.160. Statistical significance: Similar studies were documented for *F. carica* L.; Moraceae Fu’s Fs=-8.66 for ITS1, Fu’s Fs=-7.093 for 5.8 S gene, Fu’s Fs=-4.40 for ITS2 and Fu’s Fs = − 5.88, for the intergenic DNA [76]. Nevertheless the positive and non-significant values of D*: 0.9203; *p* > 0.10, F*: 0.8655; *p* > 0.10 were reported for *Phoenix dactylifera* ITS region [92]. The neutrality across the ribosomal DNA region (ITS) was greatly matched with the neutrality statistics of D Tajima, Fu, Li and Fu. The average of the pairwise nucleotide differences; k: 99.667, and Nucleotide diversity, Pi: 0.06703 was resolved for the tested *Ficus* species.

### Metabolic profiling of* Ficus* species

**Fig. 5:**
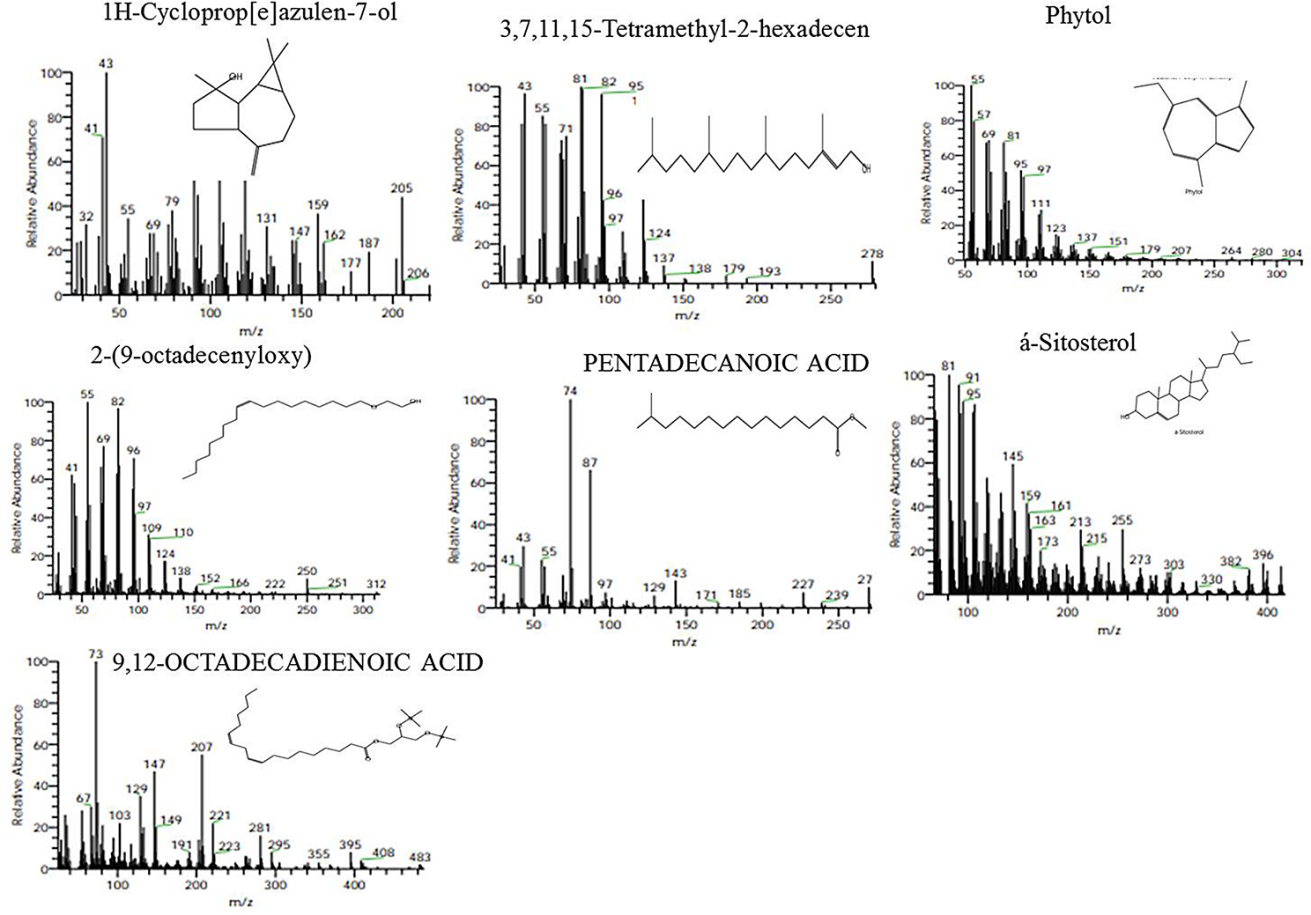
GC-MS chromatogram of the shared compounds of the methanolic extract of the experimented *Ficus* species

**Fig. 6:**
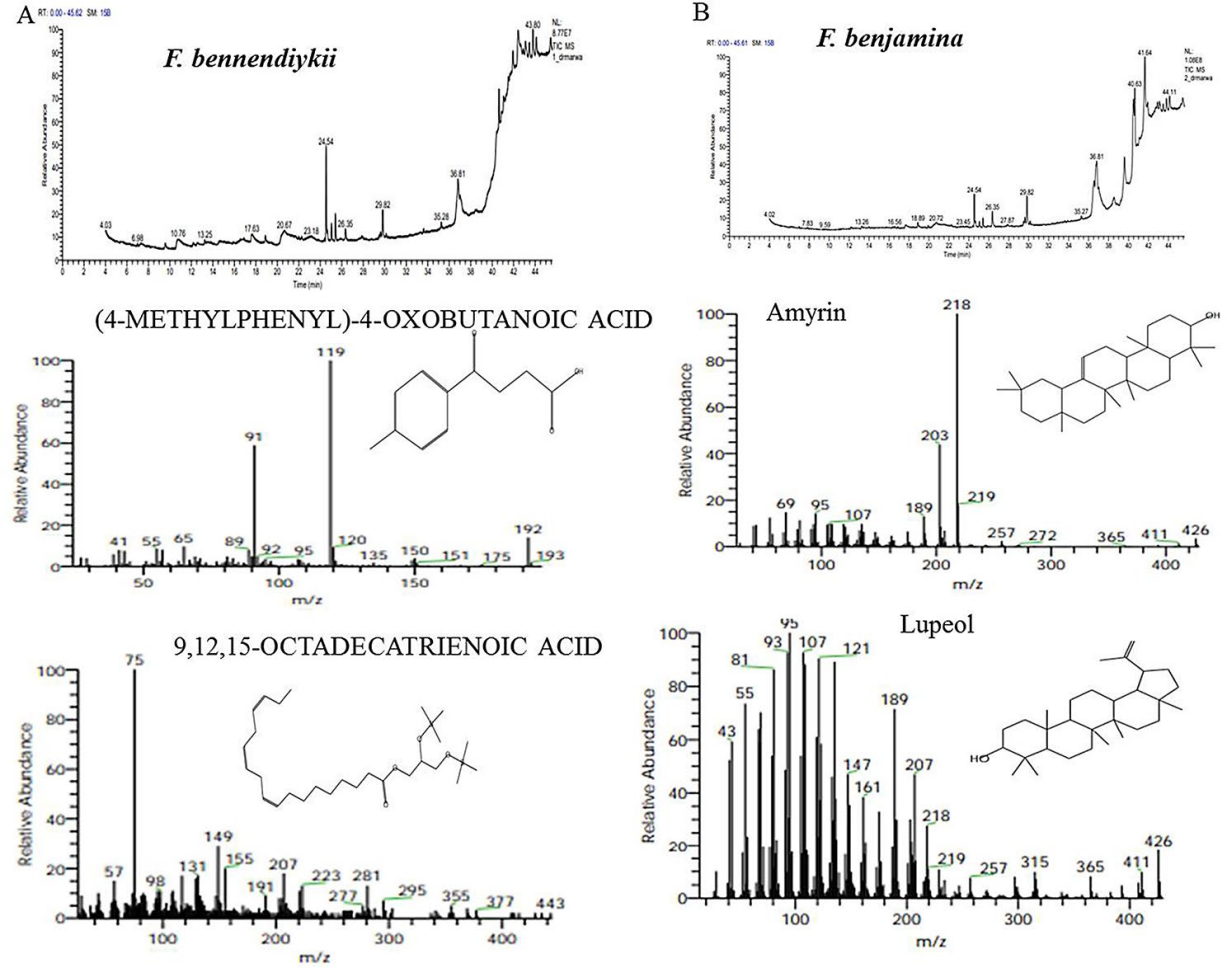
The ion current chromatogram and mass spectra chromatogram of the methanolic extracts of *F. bennendiykii* (**A**) and *F. benjamina* (**B**). The most prominent compounds of the methanolic of *F. bennendiykii* and *F. benjamina.* were shown under the ion current chromatogram

**Fig. 7:**
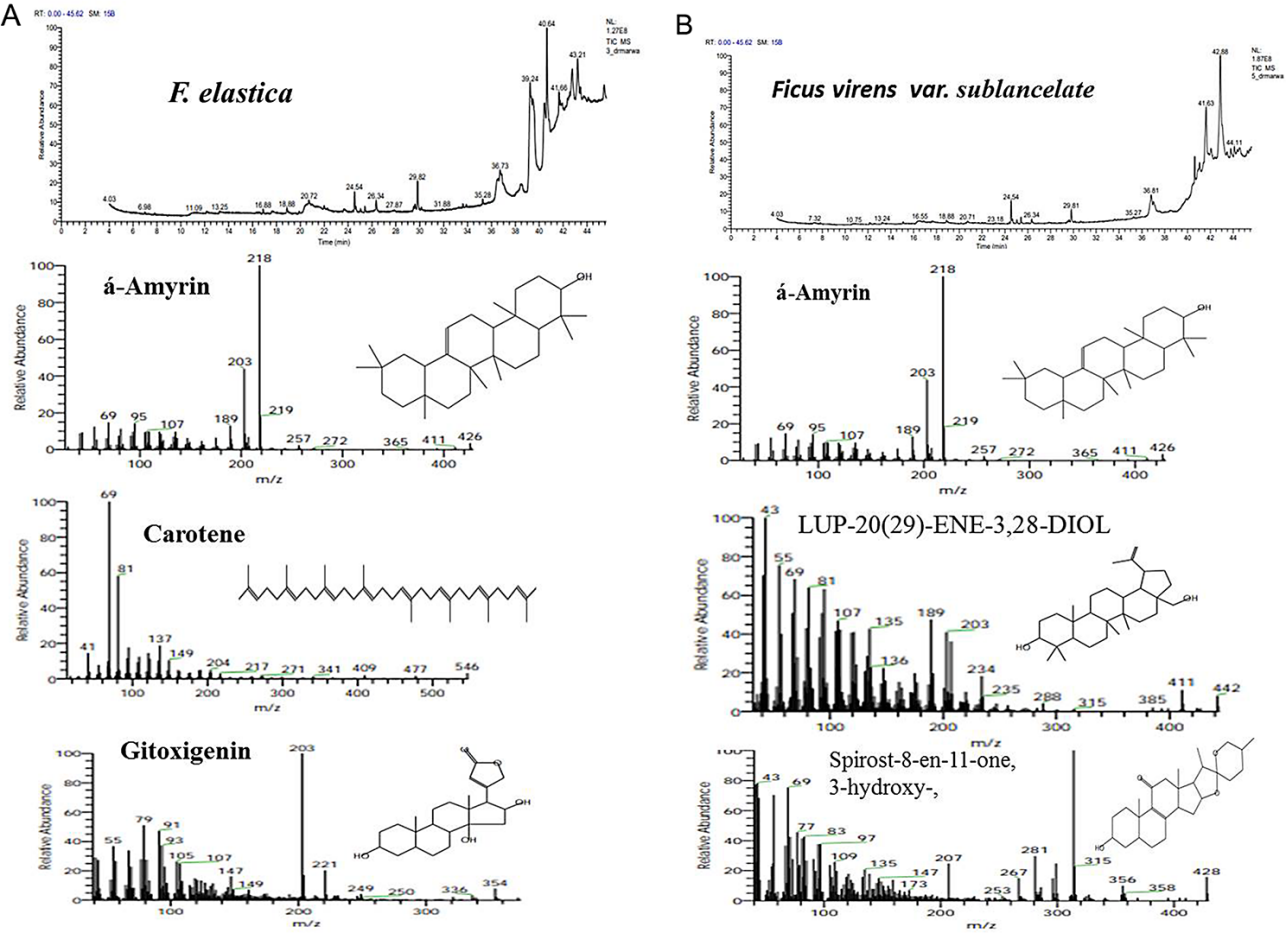
The ion current chromatogram and mass spectra chromatogram of the methanolic extracts of *F. elastica* (**A**) and *F. virens var sublancelate* (**B**). The most prominent compounds of the methanolic of *F. elastica* and *F. virens var sublancelate* were shown under the ion current chromatogram

**Fig. 8:**
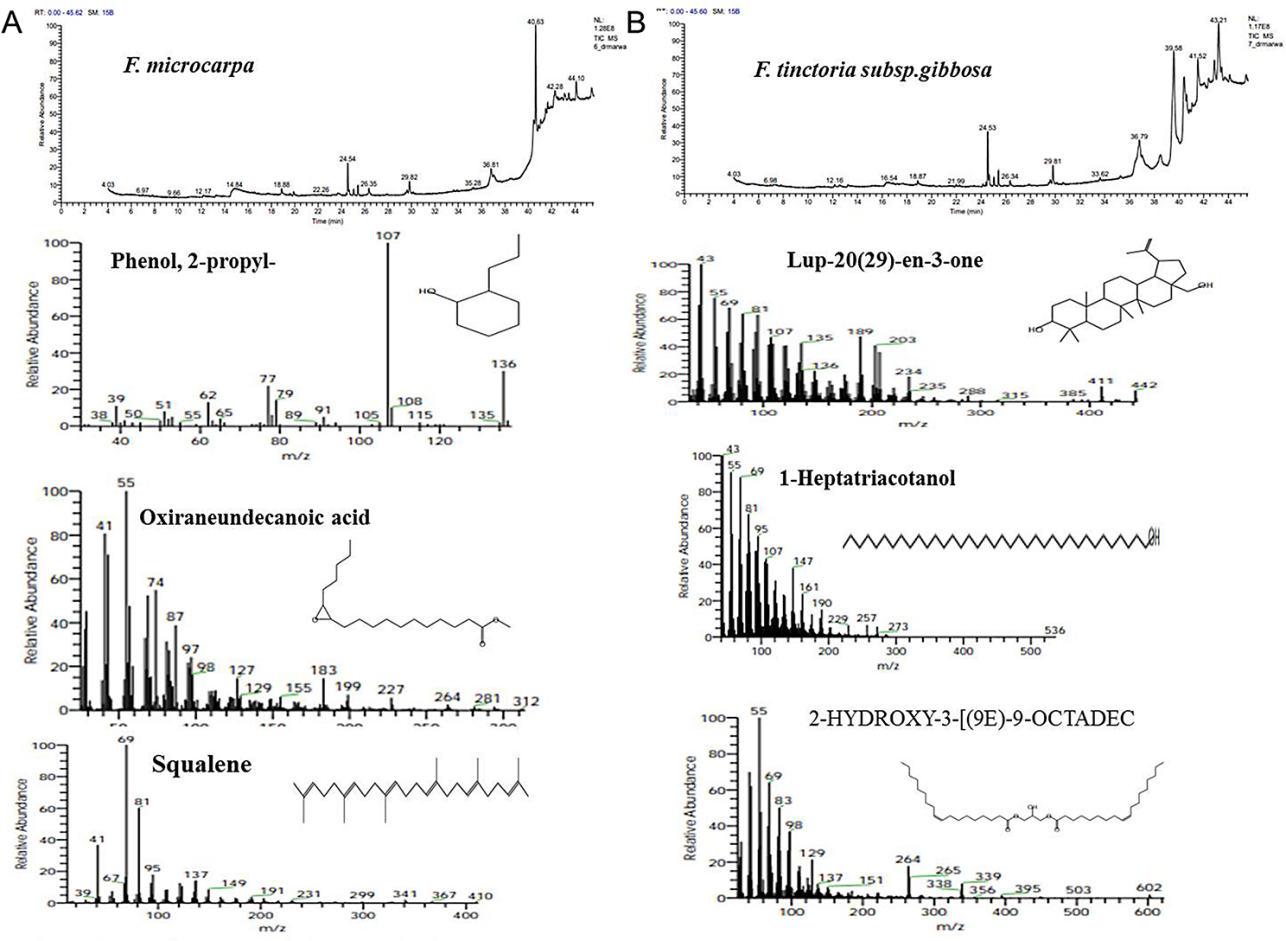
The ion current chromatogram and mass spectra chromatogram of the methanolic extracts of *F. microcarpa* (**A**) and *F. tinctoria subsp. gibbosa* (**B**). The most prominent compounds of the methanolic of *F. microcarpa* and *F. tinctoria subsp gibbosa* were shown under the ion current chromatogram

**Fig. 9:**
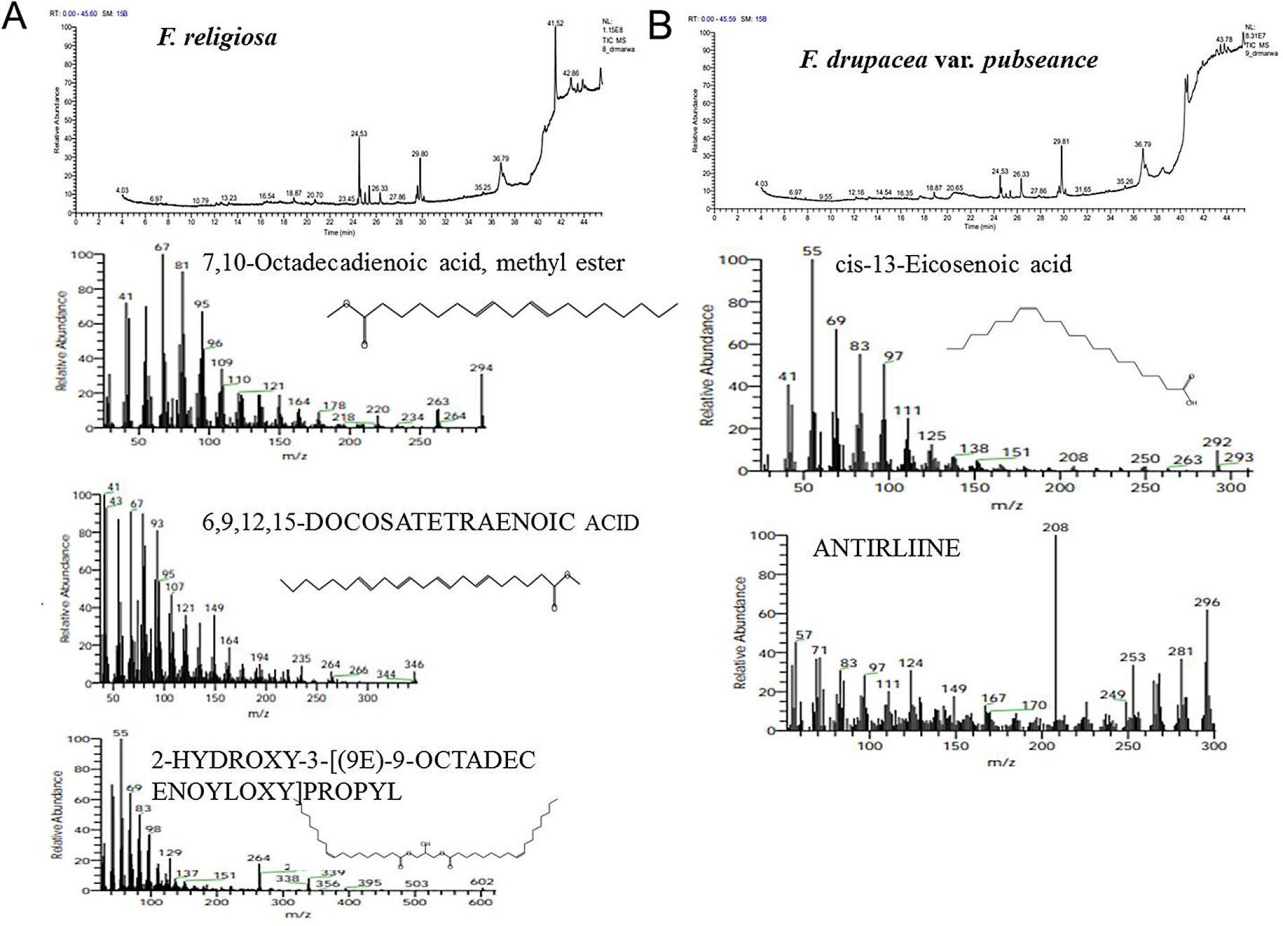
The ion current chromatogram and mass spectra chromatogram of the methanolic extracts of *F. religiosa* (**A**) and *F. drupacea var pubseance* (**b**). The most prominent compounds of the methanolic of *F. religiosa* and *F. drupacea var pubseance.* were shown under the ion current chromatogram

**Fig. 10:**
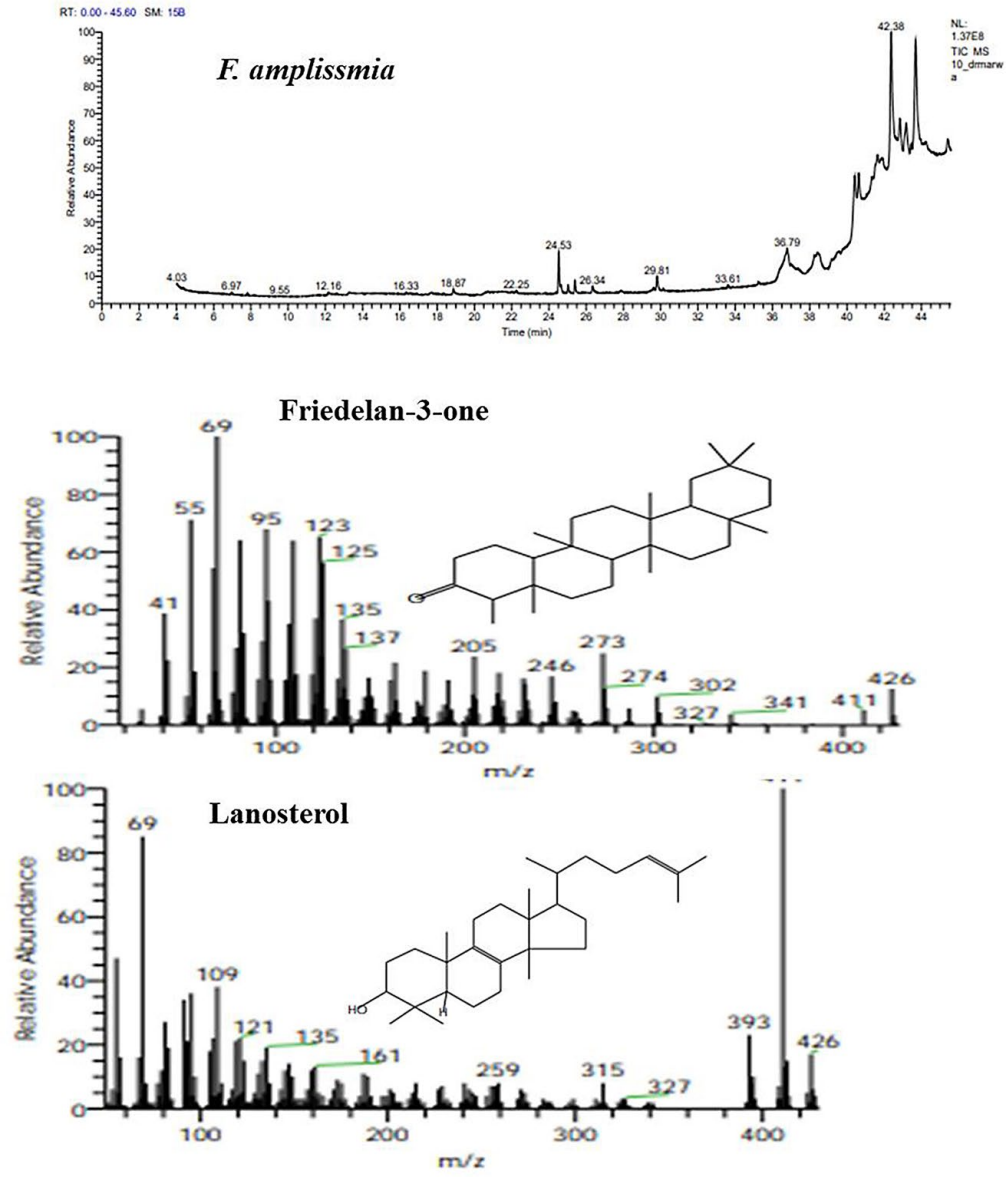
The ion current chromatogram and mass spectra chromatogram of the methanolic extracts of *F. amplissmia.* The most prominent compounds of the methanolic of *F. amplissmia* were shown under the ion current chromatogram

**Fig. 11:**
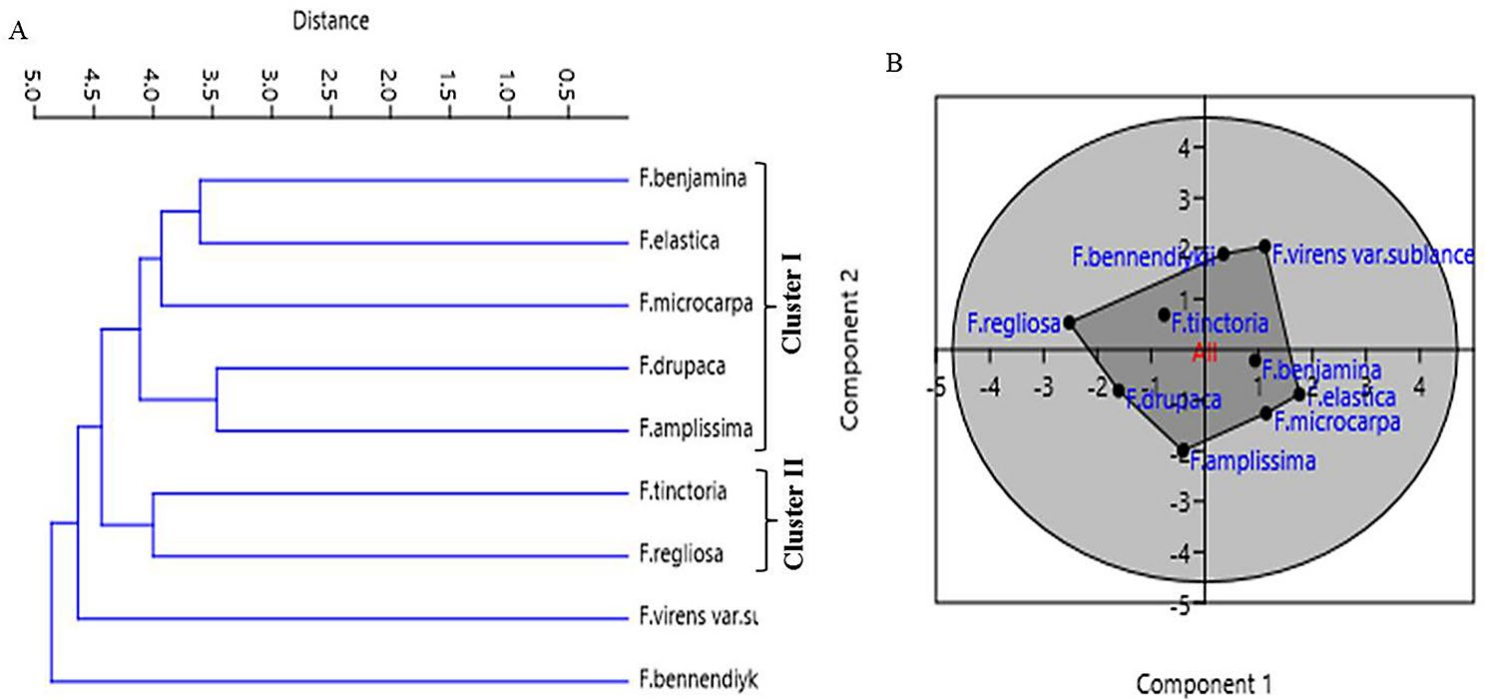
UPGMA analysis (**A**) and PCA (**B**) analysis of the experimented *Ficus* species, based on their metabolic profiling by the GC-MS analysis

The metabolic profiling of the nine species of *Ficus* species; *Ficus binnendijkii*, *F benjamina*, *F. elastic*, *F. virens* var. *sublancelata*,* F. microcarpa*,* F. tinctoria* subsp. *gibbosa*,* F. religiosa*,* F. drupacea* var. *pubescens* and *F. amplissima* was analyzed (Table 5). Metabolic profiling has been frequently used as a sophisticated chemo-taxonomic tool in plant identification that usually relied on phenotypic attributes of plants, in correlation with the gene expression-ecological interactions. Recently, chemotaxonomical analyses have been emerged as reliable tools for the classification of plants, supporting the conventional taxonomic characteristics and molecular DNA barcoding [95–102]. The volatile chemical compounds of plants have been nominated as a key technique for plant differentiation, metabolic profiling of plants, confirming the traditional taxonomical traits. The major challenge of the conventional morphometric taxonomy is a time overwhelming, reliance on plant growth stage and environmental conditions [95]. The metabolic profiling of plants has been utilized frequently for taxonomy of Convolvulaceae family [95], tree-fern *Cyathea* (Cyatheaceae) [96], *Hypericum* [97], *Solanum* spp [98], *Centaurea galicicae* and *C. tomorosii* (Asteraceae) [99, 100], subfamily Caesalpinioideae [101]. The GC-MS has been used for detection of the bioactive constituents of the plants that might be with usefulness in drug research and discovery [102–104]. The methanolic extracts of *Ficus* leaves from the taxa under investigation contained about 66 bioactive metabolites as confirmed by GC-MS analysis with an obvious fluctuation among the tested plants. The identified compounds of the studied taxa, their retention times, and concentration (area %) were presented (Table 5; Figs. 5, 6, 7, 8, 9 and 10). Thus, these compounds could be designated as the chemotaxonomic markers for the investigated taxa of in *Ficus* at the genus level unique metabolites were assigned to each plant species in addition to the common shared ones. The unique compounds might be ascribed to the biological identity of the plant, or surrounding environmental biotic and abiotic conditions, or biological identity of their endogenous microbiome, or microbiome-plant interactions. For example, the phytochemical compounds 4-(4-Methylphenyl)-4-Oxobutanoic acid, benzoic acid, hydroxy-methyle ester, 17a-Allyl-3á-acetoxy-17a-aza-D-hom-oandrost-5-ene-17-one, Tetracosanoic acid and 2-aminoethanethiol hydrogen sulfate were exclusively reported in *F. bennendiykii.* These metabolites were nominated as anti-inflammatory, anti-ulcer, anti-diabetic, antimicrobial, antimalarial, antiviral, anti-hyper-lipidaemic, anti-cancer compounds, and anti-HIV activities [105]. Several phytocompounds were assigned as an exclusive diagnostic chemical trait for each individual taxa. For instance, Gitoxigenin has been noticed as diagnostic traits of *F. elastica*, while, spirost-8-en-11-one, and 3-hydroxy were observed as diagnostic chemical for *F. virens* var *sublancelata.* The compounds phenol, 2-propyl, oxirane-undecanoic acid and squalene were reported as diagnostic features of *F. microcarpa*. These compounds were nominated with their antitumor activities, antioxidant, anti-inflammatory, and anti-atherosclerotic properties [106–112]. As well as, cis-13-Eicosenoic acid and antirliine were recognized as diagnostic traits of *F. drupacea* var *pubescens*, while, lanosterol and Friedelan-3-one was recognized as diagnostic feature of *F. amplissima*, with their common antibacterial, antifungal, anti-inflammatory, and antidiarrheal activities [113]. The compound Lup-20(29)-en-3-one has been reported with different concentrations among the *Ficus* species, *F. benjamina* (8.51%), *F. elastica* (3.91%), *F. virens* var *sublancelata* (24.12%), *F. tinctoria* subsp. *gibbosa* (25.01%) with complete absence in *F. binnendijkii*, and *F. microcarpa*. The genus *F. religiosa* is a medicinal herb that plays a significant role within medicinal herbs. All plant parts are used to make herbal medicines. Its ingredients are positioned as a crucial component in the innovative therapeutic industry [114, 115]. The clustering analysis of the studied *Ficus* species based on their metabolic profiling by GC-MS analysis was conducted by NTYSY-pc software [66]. From the GC-MS metabolic pattern of the tested nine *Ficus* spp, the taxa were categorized into two distinct clusters I and II (Fig. 11). The cluster I mainly involve *F. elastica*, *F. benjiamina*, *F. drupacea* var. *pubescens*, *F. amplissima*, which belongs to subsection Conosycea according to Berg classification [23]. While the cluster II had *F. tinctoria* subsp. *gibbosa* and *F. religiosa*. However, the *F. virens* var. *sublancelata* and *F. binnendijkii* were separated into two distinct clusters. Practically, the obvious fluctuation on the chemical identity and amounts of the metabolites among the tested plants could be related to the biological identity of the plants and their endogenous microbiome and subsequent microbiome communication and cross-talking. Since, biological identity of the microbiome of plants has a pivotal correlation with the ultimate metabolites of plants, that might be regulate the gene expression of the secondary metabolites. Thus, the compound Lup-20(29)-en-3-one could be used as a preliminary as a distinctive chemotaxonomic marker for these plants, in contrary to the other taxa. The compound Lup-20(29)-en-3-one and its derivatives have extensive pharmacological applications such as anticancer, antioxidant, anti-inflammatory and antimicrobial activities [116–118]. Nevertheless, as revealed from the metabolic profiling of the experimented *Ficus* plants, seven major compounds were shared among all the tested plant species with an obvious fluctuation. The shared compounds among the tested taxa were H-cycloprop[e]azulen-7-ol, 3,7,11,15-Tetramethyl-2-hexadecen-1-ol, 2-(9-octadecenyloxy), pentadecanoic acid, Phytol, á-Sitosterol and 9,12-octadecadienoic acid were present in the nine studied taxa of *Ficus* spp. The fluctuation of these compounds among the tested taxa might be ascribed to the environmental effect on the gene expression patterns. Thus, these compounds were designated as the chemotaxonomic markers for the investigated taxa of in the tested species of *Ficus*. Among the highest frequent compounds, phytol, 3,7,11,15-Tetramethyl-2-hexadecen-1-ol and pentadecanoic acid were recognized with their anti-inflammatory, antioxidants, anticancer, antimicrobial and diuretic [115, 116]. As well as, the compound 9,12,15-Octadecatrienoic acid have an anti-inflammatory, hypo-cholesterolemic, cancer preventive, hepatoprotective, nematicide, antihistaminic, antieczemic, antiacne, antiarthritic and antiandrogenic activities [117, 118]. Terpenoids have antibacterial, antiallergic and anti-inflammatory properties in addition to their ability to regulate metabolism and function as antioxidants, with collectively function in the plant defense [115]. The fatty acids n-hexadecanoic acid, 9,12-octadecanoic acid, and hexadecanoic acid methyl ester were usually associated with the antioxidant activity of the plant [118, 119].

**Table 5:** Metabolic profiling of the plants by GC-MS analysis

	RT	Compounds	F. bennendiykii	F. benjamina	F .elastica	F. virens	F. microcarpa	F. tinctoria	F. religiosa	F. drupacea	F. amplissima
1	4.38	2-phenyl-1,3-dioxan-5-yl ester	-	--	------------	0.13	-------------	----------	------------	----------	------------
2	6.96	Dotriacontane	0.48	------------	------------	0.15	-------------	0.21	0.33	0.50	------------
3	7.82	Cyclopentasiloxane, Decamethyl	-------	------------	------------	0.21	-------------	0.21	0.29	-------	------------
4	9.56	Benzoic acid, 2-Hydroxy-,	1.23	-----------	------------	-------	-------------	----------	------------	----------	------------
5	10.74	4-(4-Methylphenyl)-4-Oxobutanoic acid	4.49	-------------	-----------	--------	-------------	----------	------------	----------	------------
6	12.1	Spironolactone	-----------	----------	----------	0.46	-------------	----------	0.82	0.93	------------
7	12.17	17a-Allyl-3á-acetoxy-17a-aza-D-hom oandrost-5-ene-17-one	1.20	----------------	------------	----------	-------------	----------	------------	----------	------------
8	12.54	3, 5-Heptadienal, 2-Ethylidene-6-Methyl	--------------	----------------	------------	----------	-------------	----------	0.76	----------	------------
9	13.24	2-(Acetyloxy)-1-(Hydroxym Ethyl)Ethyl Acetate	0.92	----------------	------------	0.39	-------------	0.45	0.76	----------	------------
10	14.5	3-Methyl-4-(2, 6, 6-Trimethyl	--------------	----------	--------	--------	0.69	------	--------	------	-----------
11	14.7	Phenol, 2-propyl-	---------------	---------	----------	-------	2.90	--------	------------	-------	------
13	16.3	4 H-1-Benzopyran-4-One	--------------			0.80		0.59			
12	16.88	Cyclohexan-1-ol-2-carboxylic acid, 2-allyl-3-methyl-, ester	---------------	----------	0.64	-----------	--------------	---------	------	--------	------------
13	16.8	Cyclopentanedicarboxy	0.61	------------	-----------	--------	-------------	--------	------------	-------	----------
14	17.6	Tetracosanoic acid,	2.42	----------	---------	--------	------------	--------	----------	-------	-----------
15	18.8	1 H-Cycloprop[e]azulen-7-ol,	1.70	0.87	0, 83	0.66	1.64	0.65	1.23	1.91	0.87
16	19.89-	Cyclopenta[1,3]cyclopropa[1,2] cyclo hepten-3(3aH)-one	_−−−−−−−−−−−−−−−_	_−−−−−−−−−−−−−_	_−−−−−−−−−−−−−_	_−−−−−−−−−−−−_	_1.21_	_−−−−−−−−−−−−_	_0.63_	_−−−−−−−−−−−−_	_−−−−−−−−−−−−−−_
17	19.91	10,13-Octadecadiynoic Acid,	-------	0.53	------------	--------	-------------	--------	------------	---------	----------
18	20.65	2-Aminoethanethiol Hydrogen Sulfate (Ester)	1.58	------------	----------	----------	-------------	-------	------------	-------	---------
19	20.7	1,2-Longidione	------	------------	0.77	------	-------------	----------	0.95	---------	----------
20	20.71	10-Heptadecen-8-Ynoic Acid, Methyl Ester, (E)-	------	------------	------------	0.51	------------	---------	-------	-------	--------
21	22.2	Chamazulene	0.96	------------	------------	0.27	-------------	--------	------------	---------	-----------
22	24.1	Oxiraneoctanoic acid, 3-octyl	-------------	------------	-----------	-------	-----------	0.54	------------	----------	--------
23	24.3	Oleic Acid	--------	------------	----------	-------	------------	0.50	----------	--------	------------
24	24.4	Curan-17-oic acid,	0.60	----------	--------	------	-----------	---------	----------	---------	------------
25	24.5	3,7,11,15-Tetramethyl-2-hexadecen-	18.33	4.92	2.7	4.69	8.15	7.39	12.75	5.80	5.51
26	24.6	cis-13-Eicosenoic acid	--------	------------	------------	-------	-------------	----------	------------	2.82	----------
27	24.6	1-Dodecanol	1.72	0.80	0.81	1.00	1.26	1.47	2.76	----------	0.97
28	25.4	2-(9-octadecenyloxy)-, (Z)	5.24	1.39	0.75	1.29	2.49	2.06	3.57	2.03	1.78
29	26.3	Pentadecanoic acid,	2.49	2.51	1.54	0.98	1.9	0.87	2.04	5.91	0.97
30	26.3	Oxiraneundecanoic acid	--------------	------------	-----------	--------	1.94	----------	------------	----------	------------
31	27.87	2-Hydroxy-3-[(9E)-9-Octadec enoyloxy]	1.11	---------------	------------	--------	----------	----------	------------	---------------	---------------
32	29.4	7,10-Octadecadienoic acid	-----------------	----------------	------------	--------	-------------	----------	1.34	----------	------------
33	29.4	Linoleic acid ethyl ester	0.73	0.53	0.65	0.31	--------------	0.29	---------	1.18	------------
34	29.	6,9,12,15-Docosatetraenoic	-----------------	--------------	------------	------	-------------	0.85	3.78	----------	------------
35	29.61	9-Octadecenoic acid (Z)	1.50	1.24	1.11	0.67	1.19	----------	------------	3.30	------------
36	29.8	Phytol	5.81	4.40	4.13	2.81	3.40	2.70	9.01	14.55	2.07
37	30.1	Cyclopropanebutanoic acid,	---------	-------	----------	-------	--------------	0.30	------------	----------	--------
38	30.13	Methyl-9,9,10,10-D4-Octadeca noate	0.95	0.63	------------------	0.29	-------------	----------	1.30	1.73	------------
39	35.25	Androstan-17-one, 3-ethyl-3-hydroxy-, (5à)-	---------------	------------	-----------	----------	-------------	----------	0.66	1.14	------------
40	5.2	Docosahexaenoic acid,	1.71	0.58	0.84	--------	--------------	----------	------------	----------	------------
41	36.4	á-Amyrin	-------------	14.13	16.76	18.37	-------------	2.63	------------	----------	------------
42	36.7	á-Sitosterl	11.53	5.83	1.58	5.65	4.20	4.83	7.20	12.10	3.71
43	38.4	Betulin	------	------------	6.09	-------	-----------	----------	-----	--	5.30
44	38.44	9,12,15-Octadecatrienoic	6.81	----------	0.88	-------	-----------	-----	-----------	1.34	2.80
45	38.5	Gitoxigenin	------	------------	1.29	--------	-------------	----------	------------	----------	------------
46	39.41	2-Hydroxy-3-[(9E)-9-Octadec enoyloxy]propyl	-------	--------	------------	-------	------------	---------	1.21	----------	------------
48	39.5	Lup-20(29)-en-3-one	--------	8.51	3.91	24.12	-----------	25.01	------------	----------	------------
49	39.9	Trilinolein	1.07	0.42	1.04	0.7	2.03	----------	------------	----------	------------
50	40.4	Lupeol	--------	18.42	8.57	--------	12.67	9.46	------------	22.51	7.57
51	40.6	1-Heptatriacotano	1.68	---------------	2.67	--------	-------------	10.76	1.64	7.35	6.53
52	40.6	Carotene, 7, 7’, 8, 8’, 11, 11’, 12, 12’	--------------	-------------	14.76	4.73	-------------	----------	------------	----------	------------
53	40.6	Squalene	-----------------	--------------	---------	-----	25.71	----------	------------	----------	------------
54	40,8	2,3-BIS[(Trimethylsilyl)oxy ]Propyl ester	-----------------	----------------	------------	2.03	1.51	----------	------------	----------	------------
55	41.0	2-Oleoylglycerol, 2TMS derivative	--------------	1.32	----------	--------	-------------	----------	------------	1.03	0.90
56	41.05	Spirost-8-en-11-one, 3-hydroxy-,	----------------	----------------	------------	2.84	-------------	----------	------------	----------	------------
57	41.5	Antirliine	-----------------	---------------	------------	--------	-------------	----------	------------	2.71	------------
58	41.52	2-Hydroxy-3-[(9E)-9-Octadec enoyloxy]Propyl	-----------------	2.12	------------	----------	-------------	7.71	17.21	----------	------------
59	41.9	Arabinitol, Pentaacetate	-----------------	1.81	0.72	0.30	4.81	5.91	0.55	2.05	5.81
60	42.3	Lanosterol	----------------	-------------	----------	-------	-------------	----------	----------	----------	19.86
61	42.5	Rhodopin	---------------	------------	0.65	------	-------------	----------	-----------	----------	------------
63	43.45	9,12-Octadecadienoic acid	7.29	2.56	1,41	1,20	5.64	4.07	4.68	1.78	1.03
64	43.6	Friedelan-3-one	----------------	--------------	-----------	-------	-------------	----------	-----------	----------	18.40
65	43.7	3,5-Dihydroxy-7-Methoxy	-------------	-------------	------------	2.85	----------	----------	------------	----------	----------
66	45.4	Ursodeoxycholic acid	----------------	--------------	2.8	2.23	3.62	----------	-----------	----------	2.54

## Conclusion

Nine taxa of the *Ficus* species namely; F. *binnendijkii*,* F. benjamina*,* F. elastica*,* F. virens* var. *sublancelate*,* F. microcarpa*,* F. tinctoria* subsp. *gibbosa*,* F. religiosa*,* F. drupacea* var. *pubescens* and *F. amplissima*, have been selected for revising, their taxonomical features, based on anatomical features, the DNA barcoding and metabolic profiling traits. From anatomical features, molecular barcoding, and metabolic profiling, the studied nine taxa of *Ficus* were categorized into two distinct clusters I and II. The cluster I mainly involve *F. elastica*, *F. benjiamina*, and *F. drupacea* var. *pubescens*, which belongs to subsection *Conosycea*. The cluster II had *F. tinctoria* subsp. *gibbosa* and *F. religiosa*, while, the cluster III involve *F. virens* var *sublancelata* and *F. binnendijkii*. Thus, from the morphological, molecular traits and metabolic profiling by GC-MS analysis, the studied *Ficus* species were clearly delineated. Decisively, the morphological features, molecular barcoding using ITS sequences, and metabolic traits by GC-MS analysis, authenticate the taxonomical diversity of the species of the genus *Ficus*.

### Authors’ contributions

M.M.E conceived, and wrote the manuscript. A.S.A.E revises and edits the manuscript. A.M.S and S.S.T perform the anatomical analysis. H.H.E revised the manuscript. All authors read and approved the manuscript.

## Electronic supplementary material

Below is the link to the electronic supplementary material.

Supplementary Material 1

Supplementary Material 2

## Data Availability

All datasets generated for this study are included in the article. The ITS sequences of the experimented plants were deposited on Genbank with accession numbers as follows: https://www.ncbi.nlm.nih.gov/nuccore/ON552478https://www.ncbi.nlm.nih.gov/nuccore/OP597689https://www.ncbi.nlm.nih.gov/nuccore/ON526736https://www.ncbi.nlm.nih.gov/nuccore/OP374191https://www.ncbi.nlm.nih.gov/nuccore/ON525756https://www.ncbi.nlm.nih.gov/nuccore/ON514265https://www.ncbi.nlm.nih.gov/nuccore/OP363205https://www.ncbi.nlm.nih.gov/nuccore/ON514560https://www.ncbi.nlm.nih.gov/nuccore/OP363202.
