# Supplementary material for: DNA barcoding, micromorphology and metabolic traits of selected Ficus L. (Moraceae) species from Egypt
Source: BMC Plant Biol. 2024 Nov 13;24:1067. doi: 10.1186/s12870-024-05683-4 (PMC11559249; doi:10.1186/s12870-024-05683-4)
Supplement: Supplementary file 1 — Supplementary Material 1 [file 12870_2024_5683_MOESM1_ESM.docx]

Table S1: Anatomical features of the studied *Ficus* species

| **Characters / stats**  **Species** | | | ***1. F.amplissima*** | ***2. F.benjamina*** | ***3. F.binnendijkii*** | ***4. F.drupacea v.pubescens*** | ***5. F.elasctica*** | ***6. F.microcarpa*** | ***7. F.religiosa*** | ***8. F.tinctoria subsp.gibbosa*** | ***9. F.virens v.sublanceolata*** |
| --- | --- | --- | --- | --- | --- | --- | --- | --- | --- | --- | --- |
| **Lamina Anatomy** | **Midrib outline** | | | | | | | | | | |
|  |  | Rounded | 1 | 1 | 1 | 1 | 0 | 0 | 1 | 1 | 1 |
|  |  | Arc shaped | 0 | 0 | 0 | 0 | 1 | 0 | 0 | 0 | 0 |
|  |  | Line | 0 | 0 | 0 | 0 | 0 | 1 | 0 | 0 | 0 |
|  | **Intercostal rejoin level** | | | | | | | | | | |
|  |  | Convex | 1 | 1 | 0 | 1 | 0 | 0 | 1 | 1 | 1 |
|  |  | Concave | 0 | 0 | 1 | 0 | 0 | 0 | 0 | 0 | 0 |
|  |  | Line | 0 | 0 | 0 | 0 | 1 | 1 | 0 | 0 | 0 |
|  | **Cuticle thickness** | | | | | | | | | | |
|  |  | Thin | 1 | 1 | 0 | 0 | 0 | 1 | 1 | 1 | 1 |
|  |  | Thick | 0 | 0 | 1 | 1 | 1 | 0 | 0 | 0 | 0 |
|  | **Cuticle surface** | | | | | | | | | | |
|  |  | Smooth | 1 | 0 | 1 | 1 | 1 | 1 | 1 | 1 | 1 |
|  |  | Warty | 0 | 1 | 0 | 0 | 0 | 0 | 0 | 0 | 0 |
|  | **Epidermal cells shape** | | | | | | | | | | |
|  |  | Oblong | 1 | 1 | 1 | 1 | 1 | 1 | 1 | 1 | 1 |
|  |  | cubic | 0 | 0 | 1 | 1 | 1 | 1 | 1 | 1 | 1 |
|  |  | Babbli | 1 | 1 | 1 | 0 | 1 | 1 | 1 | 1 | 1 |
|  | **Upper Epidermis layers** | | | | | | | | | | |
|  |  | Uniseriate | 1 | 1 | 1 | 1 | 1 | 1 | 1 | 1 | 1 |
|  |  | Biseriate | 0 | 0 | 0 | 0 | 0 | 0 | 0 | 0 | 0 |
|  | **Lower** **Epidermis** **layers** | | | | | | | | | | |
|  |  | Uniseriate | 1 | 1 | 1 | 1 | 0 | 1 | 1 | 1 | 1 |
|  |  | Biseriate | 0 | 0 | 0 | 0 | 1 | 0 | 0 | 0 | 0 |
|  | **Upper Epidermis** **thickness** | | | | | | | | | | |
|  |  | Thick (8-17 µm) | 0 | 0 | 0 | 0 | 0 | 0 | 1 | 0 | 1 |
|  |  | Thin (2-7 µm) | 1 | 1 | 1 | 1 | 1 | 1 | 0 | 1 | 0 |
|  | **Lower** **Epidermis** **thickness** | | | | | | | | | | |
|  |  | Thin (2-7 µm) | 1 | 1 | 1 | 1 | 1 | 1 | 1 | 1 | 1 |
|  | **Hypodermis layers** | | | | | | | | | | |
|  |  | absent | 0 | 0 | 0 | 0 | 0 | 0 | 1 | 0 | 1 |
|  |  | 1- 4 adaxial | 1 | 1 | 1 | 1 | 1 | 1 | 0 | 1 | 0 |
|  |  | 1 abaxial | 1 | 0 | 1 | 1 | 1 | 1 | 0 | 0 | 0 |
|  | **Cystoliths presence**/**location** | | | | | | | | | | |
|  |  | Present adaxial | 1 | 1 | 1 | 1 | 1 | 1 | 0 | 0 | 0 |
|  |  | Present abaxial | 0 | 0 | 0 | 0 | 0 | 0 | 1 | 1 | 1 |
|  | **Midrib ground tissue** | | | | | | | | | | |
|  |  | Parenchyma only | 0 | 1 | 0 | 0 | 0 | 0 | 0 | 0 | 0 |
|  |  | Parenchyma / collenchyma / sclerenchyma | 1 | 0 | 0 | 1 | 1 | 1 | 1 | 1 | 1 |
|  |  | Parenchyma / sclerenchyma | 0 | 0 | 1 | 0 | 0 | 0 | 0 | 0 | 0 |
|  |  | Stone cells | 0 | 0 | 0 | 0 | 0 | 0 | 0 | 1 | 0 |
|  | **Steel shape** | | | | | | | | | | |
|  |  | Crescent | 1 | 0 | 0 | 0 | 1 | 1 | 1 | 0 | 0 |
|  |  | Cycle | 0 | 1 | 1 | 1 | 0 | 0 | 0 | 1 | 1 |
|  | **Vascular bundle** **type** | | | | | | | | | | |
|  |  | Collateral | 1 | 1 | 1 | 1 | 1 | 1 | 1 | 1 | 1 |
|  | **Medullary vascular bundle in Midrib** | | | | | | | | | | |
|  |  | V. B. Presence | 0 | 1 | 1 | 1 | 1 | 1 | 1 | 1 | 1 |
|  | **Phloem patches in Midrib center** | | | | | | | | | | |
|  |  | Presence | 0 | 1 | 0 | 0 | 0 | 0 | 0 | 0 | 0 |
|  | **Mesophyll type** | | | | | | | | | | |
|  |  | Isolateral | 0 | 0 | 1 | 1 | 1 | 0 | 0 | 0 | 1 |
|  |  | Dorsiventral | 1 | 1 | 0 | 0 | 0 | 1 | 1 | 1 | 0 |
|  | **Palisade continuity at midrib** | | | | | | | | | | |
|  |  | Discontinues | 1 | 1 | 1 | 1 | 1 | 1 | 1 | 1 | 1 |
|  | Trichomes | | | | | | | | | | |
|  |  | absent | 0 | 1 | 1 | 0 | 1 | 1 | 1 | 1 | 1 |
|  |  | Unicellular/glandular | 1 | 0 | 0 | 0 | 0 | 0 | 0 | 0 | 0 |
|  |  | Unicellular/non-glandular | 0 | 0 | 0 | 1 | 0 | 0 | 0 | 0 | 0 |
|  | Crystals | | | | | | | | | | |
|  |  | Druses | 0 | 0 | 1 | 1 | 0 | 1 | 1 | 1 | 0 |
|  | Starch grains | | | | | | | | | | |
|  |  | Presence | 0 | 0 | 1 | 0 | 0 | 1 | 0 | 0 | 0 |
