# Supplementary material for: DNA barcoding, micromorphology and metabolic traits of selected Ficus L. (Moraceae) species from Egypt
Source: BMC Plant Biol. 2024 Nov 13;24:1067. doi: 10.1186/s12870-024-05683-4 (PMC11559249; doi:10.1186/s12870-024-05683-4)
Supplement: Supplementary file 2 — Supplementary Material 2 [file 12870_2024_5683_MOESM2_ESM.pptx]

## Slide 1
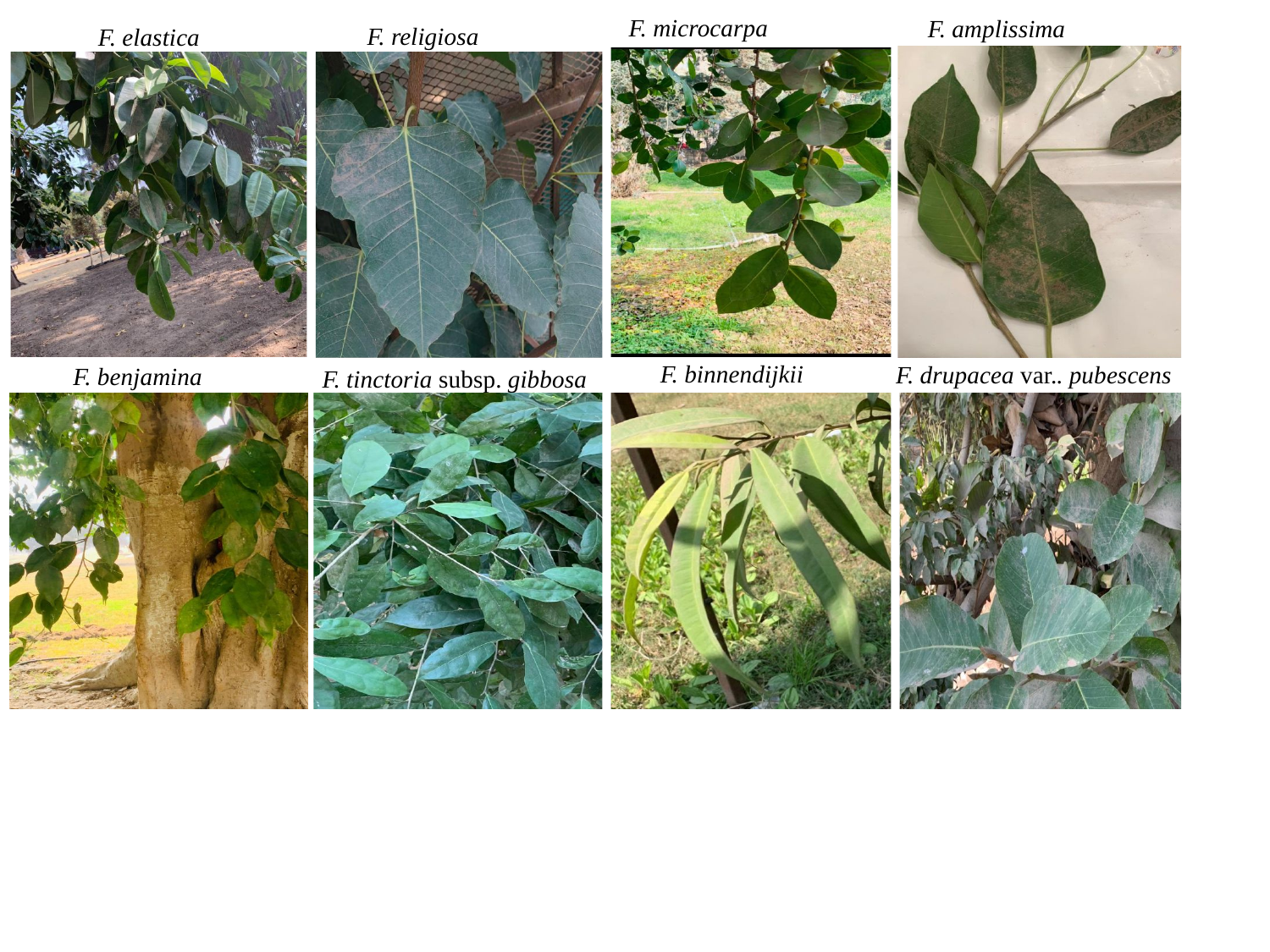

F. microcarpa
F. amplissima
F. religiosa
F. elastica
F. binnendijkii
F. drupacea var.. pubescens
F. benjamina
F. tinctoria subsp. gibbosa

## Slide 2
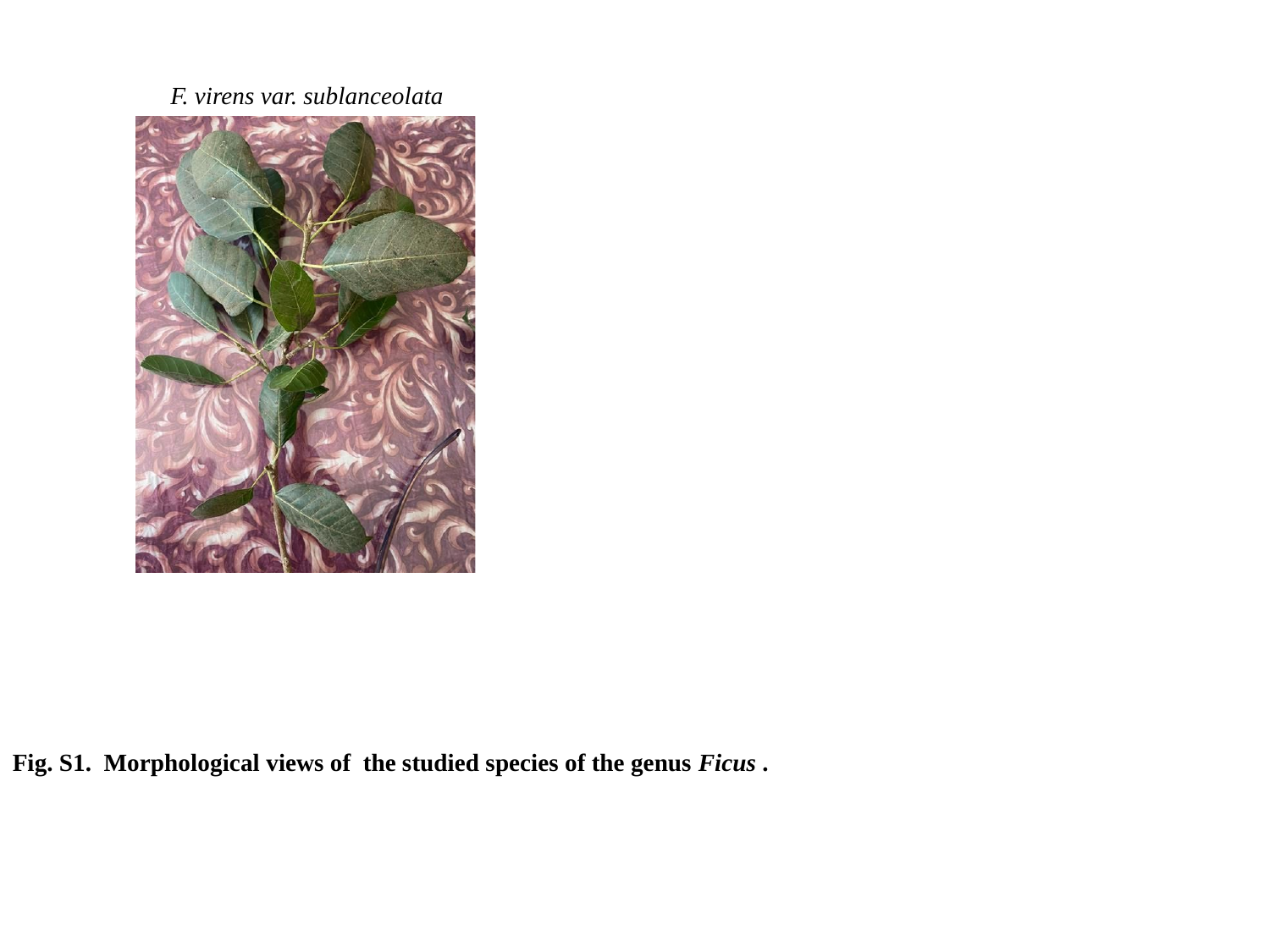

F. virens var. sublanceolata
Fig. S1. Morphological views of the studied species of the genus Ficus .
